# Single‐Cell Sequencing‐Guided Annotation of Rare Tumor Cells for Deep Learning‐Based Cytopathologic Diagnosis of Early Lung Cancer

**DOI:** 10.1002/advs.202416921

**Published:** 2025-04-15

**Authors:** Yichun Zhao, Ruoran Qiu, Zhuo Wang, Yunyun Li, Xu Yang, Yanlin Li, Xiaohan Shen, Yun Liu, Ziqiang Chen, Qihan You, Qihui Shi

**Affiliations:** ^1^ Key Laboratory of Whole‐Period Monitoring and Precise Intervention of Digestive Cancer (SMHC) Minhang Hospital and Institutes of Biomedical Sciences Fudan University Shanghai 200032 China; ^2^ Department of Pathology The First Affiliated Hospital (Qingchun campus) Zhejiang University School of Medicine Hangzhou 310003 China; ^3^ Department of Pathology The First Affiliated Hospital (Yuhang campus) Zhejiang University School of Medicine Hangzhou 311121 China; ^4^ Department of Pathology Shandong Cancer Hospital and Institute Shandong First Medical University and Shandong Academy of Medical Sciences Jinan 250117 China; ^5^ MOE Key Laboratory of Metabolism and Molecular Medicine Department of Biochemistry and Molecular Biology School of Basic Medical Sciences and Shanghai Xuhui Central Hospital Fudan University Shanghai 200032 China; ^6^ Shanghai Engineering Research Center of Biomedical Analysis Reagents Fudan Zhang Jiang Institute Shanghai 201203 China

**Keywords:** deep learning, exfoliated tumor cells, lung cancer, single‐cell sequencing

## Abstract

Deep learning (DL) models for medical image analysis are majorly bottlenecked by the lack of well‐annotated datasets. Bronchoalveolar lavage (BAL) is a minimally invasive procedure to diagnose lung cancer, but BAL cytology suffers from low sensitivity. The success of DL in BAL cytology is rare due to the rarity of exfoliated tumor cells (ETCs) and their subtle morphological differences from normal cells. Single‐cell DNA sequencing (scDNA‐Seq) is utilized as an objective ground truth of ETC annotation for generating an unbiased, accurately annotated dataset comprising 580 ETCs and 1106 benign cells from BAL cytology slides. A DL model is developed, to distinguish ETC from benign cells in BAL fluid, achieving an Area Under the Curve of 0.997 and 0.956 for detecting large‐ and small‐sized ETCs, respectively. The model is applied in a discovery cohort (n = 156) to establish BAL‐based cytopathologic diagnostic model for lung cancer. The model is evaluated in a validation cohort (n = 158), and yielded 47.6% sensitivity and 97.7% specificity in lung cancer diagnosis, outperforming cytology with improved sensitivity (47.6% vs 19.0%). In an external validation cohort (n = 141), the model achieved 60.0% sensitivity and 92.5% specificity in lung cancer diagnosis.

## Graphical abstract

This study presents an innovative method to generate high‐quality, objectively annotated cytology datasets without relying on expert involvement. Large‐scale single‐cell DNA sequencing is used as an objective ground truth of cell annotation for generating an accurate, unbiased dataset of exfoliated tumor cells from bronchoalveolar lavage cytology slides. A deep learning model is developed for cytopathologic diagnosis of early lung cancer.

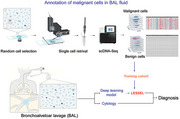

## 1 Introduction

Deep learning (DL), as a type of artificial intelligence (AI) algorithm, is characterized by its ability to learn complex representations and has been widely investigated in medical image classification and object detection for disease detection and diagnosis.^[^ ^1^ ^]^ Although DL has been shown to match or surpass human experts in some medical image analysis tasks,^[^ ^1^, ^2^ ^]^ the further improvement of DL models is majorly bottlenecked by the lack of large‐sized and well‐annotated datasets which require a large number of experts with advanced skills. Even expert‐generated annotated datasets can still be highly subjective, with different experts potentially making different judgments on the same image. Although unsupervised and semi‐supervised learning models have received extensive attention,^[^ ^1^ ^]^ these models still show performance gaps compared to supervised learning models trained on large and accurately annotated datasets. Thus, generating high‐quality, objectively annotated datasets for DL model training without relying on expert involvement remains a critical challenge.

In the field of pathologic/cytopathologic diagnosis, success of DL has been mostly shown in pathology, abrasive (e.g., cervical scraping) or aspiration cytology that contain abundant tumor cells for accurate annotation,^[^ ^3^, ^4^, ^5^, ^6^, ^7^, ^8^, ^9^ ^]^ but is rare in cytopathologic diagnosis of body fluids (e.g., urine) because of low abundance of exfoliated tumor cells (ETCs) and substantial overlap in cytologic features between ETCs and normal cells.^[^ ^3^, ^4^ ^]^ Even experts often struggle to efficiently and accurately distinguish ETCs from normal cells. The major bottleneck for DL‐based cytopathologic diagnosis is the lack of accurately annotated ETC datasets from cytopathologists.^[^ ^3^, ^4^ ^]^ To address this challenge, single‐cell DNA sequencing (scDNA‐Seq) is used as an objective ground truth of ETC annotation, instead of subjective annotation by cytopathologists, to generate an accurate and unbiased ETC dataset from Papanicolaou (PAP)‐stained cytology slides for training DL models.

Somatic copy number alterations (CNAs) are known to be present in a majority of solid tumors but occur sporadically in benign tissues.^[^ ^10^, ^11^, ^12^, ^13^ ^]^ In our previous studies, concordant single‐cell CNA profiles have been found in ETCs or circulating tumor cells (CTCs) from different types of body fluids (e.g., blood, urine, bile, pleural effusion, and ascites) and across many cancer types.^[^ ^14^, ^15^, ^16^, ^17^, ^18^, ^19^, ^20^, ^21^ ^]^ Concordant CNA profiles among multiple cells are characteristic of clonal expansion in malignant cells and absent in normal cells, leading to a quantitative criterion for determining cell malignancy with high accuracy.^[^ ^19^ ^]^ Meanwhile, we have developed a Tn5 transposase‐based method that significantly decreased the cost of scDNA‐Seq.^[^ ^20^ ^]^ Thus, low‐cost, large‐scale scDNA‐Seq provides an objective method for accurate and unbiased ETC annotation, minimizing inaccurate or incomplete ETC annotation by identification of subtle morphological features.

**Figure 1:**
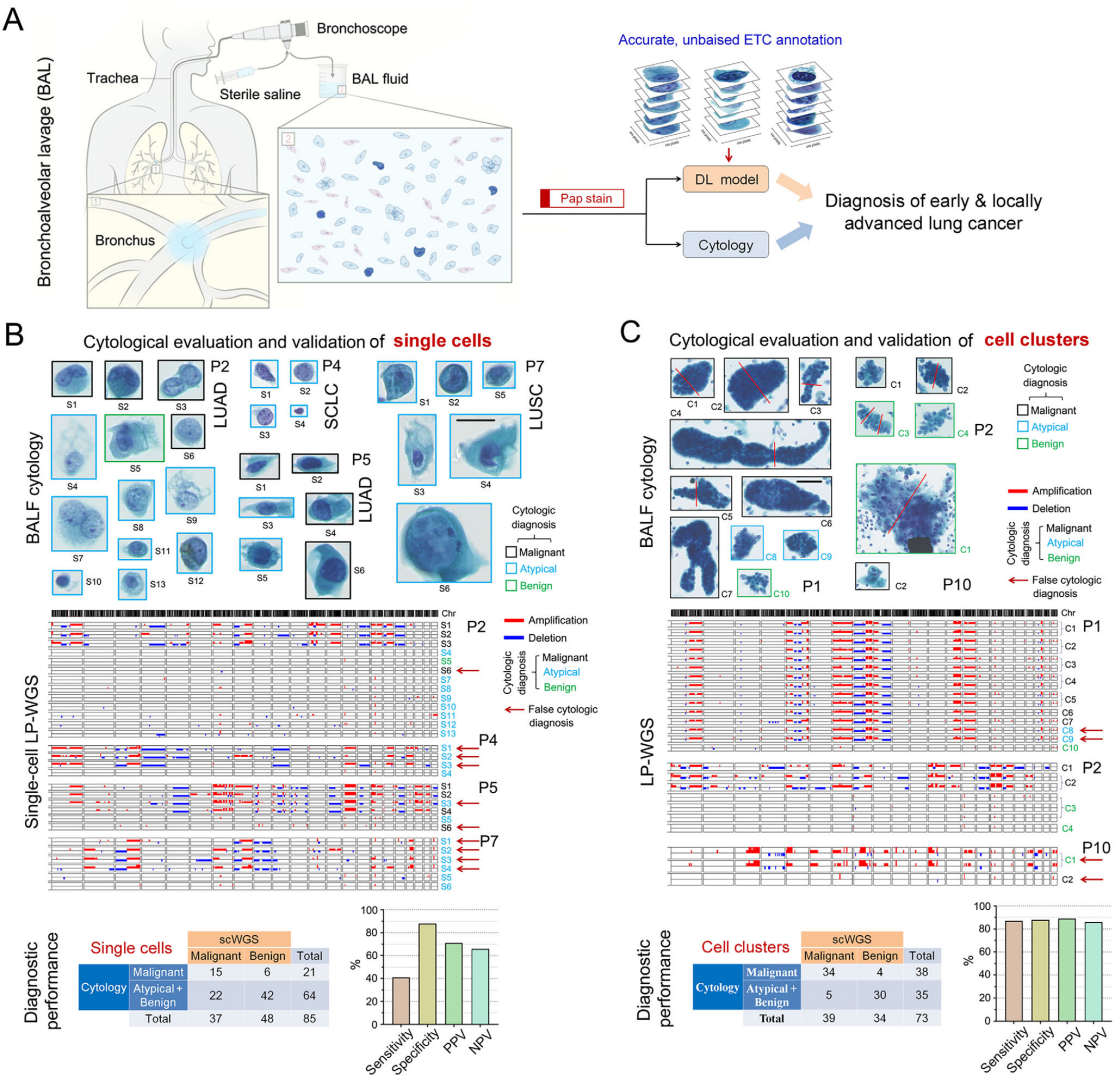
ScDNA‐Seq evaluation of cytological annotation of single cells and cell clusters from BALF. A) Schematic illustration of Pap‐stained BAL cytology‐based diagnosis and the DL model for lung cancer diagnosis. B) Pap‐stained cytology images (top) and single‐cell CNA profiles (middle) of cells annotated by expert cytologists from BALF specimens, as well as diagnostic performance of BAL cytology in diagnosing single cells using scDNA‐Seq as the ground truth (bottom). BAL cytology was dichotomized as diagnostic for benignness with atypical or benign annotation and diagnostic for malignancy with malignant annotation. Scale bar: 20 µm. C) Cytology images (top) and CNA profiles (middle) of clusters annotated by expert cytologists from BALF specimens, as well as diagnostic performance of BAL cytology in diagnosing cell clusters (bottom). Some cell clusters were cut into two or three clusters for parallel scDNA‐Seq. Scale bar: 50 µm. LUAD, lung adenocarcinoma; LUSC, lung squamous cell carcinoma; SCLC, small‐cell lung cancer; PPV, positive predictive value; NPV, negative predictive value.

Lung cancer is the leading cause of cancer‐related deaths worldwide, with early diagnosis being crucial for improving patient outcomes.^[^ ^22^, ^23^ ^]^ The growing use of chest computed tomography (CT) has led to an exponential increase in the detection of indeterminate pulmonary nodules (IPNs) suspicious for lung cancer. Although the large majority of IPNs are benign, invasive bronchoscopic biopsy or CT‐guided transthoracic needle biopsy is required for effective tissue diagnosis, leading to a large number of unnecessary invasive biopsies with attendant morbidity and rare mortality.^[^ ^24^, ^25^ ^]^ Bronchoalveolar lavage (BAL) is a minimally invasive procedure that involves the instillation of sterile saline into a subsegment of the lung, followed by suction and collection of the instilled fluid for analysis. Due to its proximity to lung tumors, BAL fluid (BALF) containing ETCs and tumor‐derived cell‐free DNA (cfDNA) represents a precious source of liquid biopsy from the lower respiratory tract and alveolar spaces of the lung (**Figure** **1**A). BAL cytology identifies ETCs/ETC clusters in BALF for diagnosing lung cancer but suffers from low diagnostic sensitivity (≈14.7%).^[^ ^26^, ^27^ ^]^ A BAL cfDNA‐based classifier has been developed for diagnosing malignant lung lesions with a 69% sensitivity in a small 35‐lung cancer patient cohort, outperforming the 12% sensitivity of BAL cytology.^[^ ^28^ ^]^ However, this 11‐gene cfDNA classifier lacks independent validation, and the cfDNA analysis is time‐consuming and costly for clinical use.^[^ ^28^ ^]^

To address the technical challenge of DL and unmet clinical need in BAL‐based lung cancer diagnosis, we developed a DL model on BAL cytology, LESSEL (Lung cancer detection with single‐cell sequencing and deep learning), for detecting ETCs in BALF based on an unbiased, accurately annotated ETC dataset generated from large‐scale scDNA‐Seq. This innovative DL model enables BAL‐based cytopathologic diagnosis of early and locally advanced lung cancer, significantly outperforming BAL cytology in diagnostic accuracy.

## 2 Results

### 2.1 ScDNA‐Seq Accurately Detects ETCs, Surpassing BAL Cytology

BALF contains a low number of ETCs and abundant inflammatory cells (e.g., alveolar macrophages).^[^ ^29^ ^]^ We first assessed the accuracy of BAL cell annotation by expert cytopathologists using single‐cell low‐pass whole genome sequencing (LP‐WGS) as the objective ground truth. Single‐cell LP‐WGS surveyed genome‐wide CNAs of annotated cells, and multiple cells with concordant single‐cell CNA profiles were identified as malignant cells based on the criterion established in our previous study.^[^ ^19^ ^]^ A total of 85 single cells from BALF samples of 8 lung cancer patients (Table S1, Supporting Information) were annotated by an expert cytopathologist, comprising 21 malignant, 64 atypical, or benign cells, and then individually retrieved for single‐cell LP‐WGS. Figure 1B shows microscopic images, cytological evaluation and CNA profiles of 29 single cells from patients P2, P4, P5, and P7. Malignant cells exhibited concordant CNA profiles, whereas normal cells were absent of CNAs. Using scDNA‐Seq as the ground truth, the diagnostic sensitivity, specificity, positive predictive value (PPV), and negative predictive value (NPV) of cytological evaluation of single cells in BALF were 40.5% (95% CI: 24.8–57.9%), 87.5% (95% CI: 74.8–95.3%), 71.4% (95% CI: 51.8–85.3%), and 65.6% (95% CI: 58.9–71.8%), respectively (Figure S1, Table S2, Supporting Information). The suboptimal performance of cytological evaluation in single ETC detection resulted in limited diagnostic sensitivity of BAL cytology in lung cancer diagnosis, which could be attributed to the limitation of subtle morphological feature recognition by cytopathologists.

In addition to single ETCs, ETC clusters are known to be present in a fraction of BALF specimens. Cytological evaluation of 73 ETC clusters from BALF samples of 17 lung cancer patients demonstrated 87.2% (95% CI: 72.5–95.7%) sensitivity, 88.2% (95% CI: 72.6–96.7%) specificity, 89.5% (95% CI: 77.1–95.6%) PPV and 85.7% (95% CI: 72.4–93.2%) NPV using scDNA‐Seq as the reference standard (Figure 1C; Figures S2 and S3 and Table S3, Supporting Information), which significantly exceeded the sensitivity of cytological detection for single ETCs. Thus, the presence of ETC clusters in BALF enhances cytopathologic diagnosis of malignancy, yet it remains a significant challenge for cytopathologists to accurately diagnose BALF specimens that lack cytologically detectable ETC clusters.

### 2.2 Generation of the Unbiased, scDNA‐Seq‐Confirmed ETC Dataset

**Figure 2:**
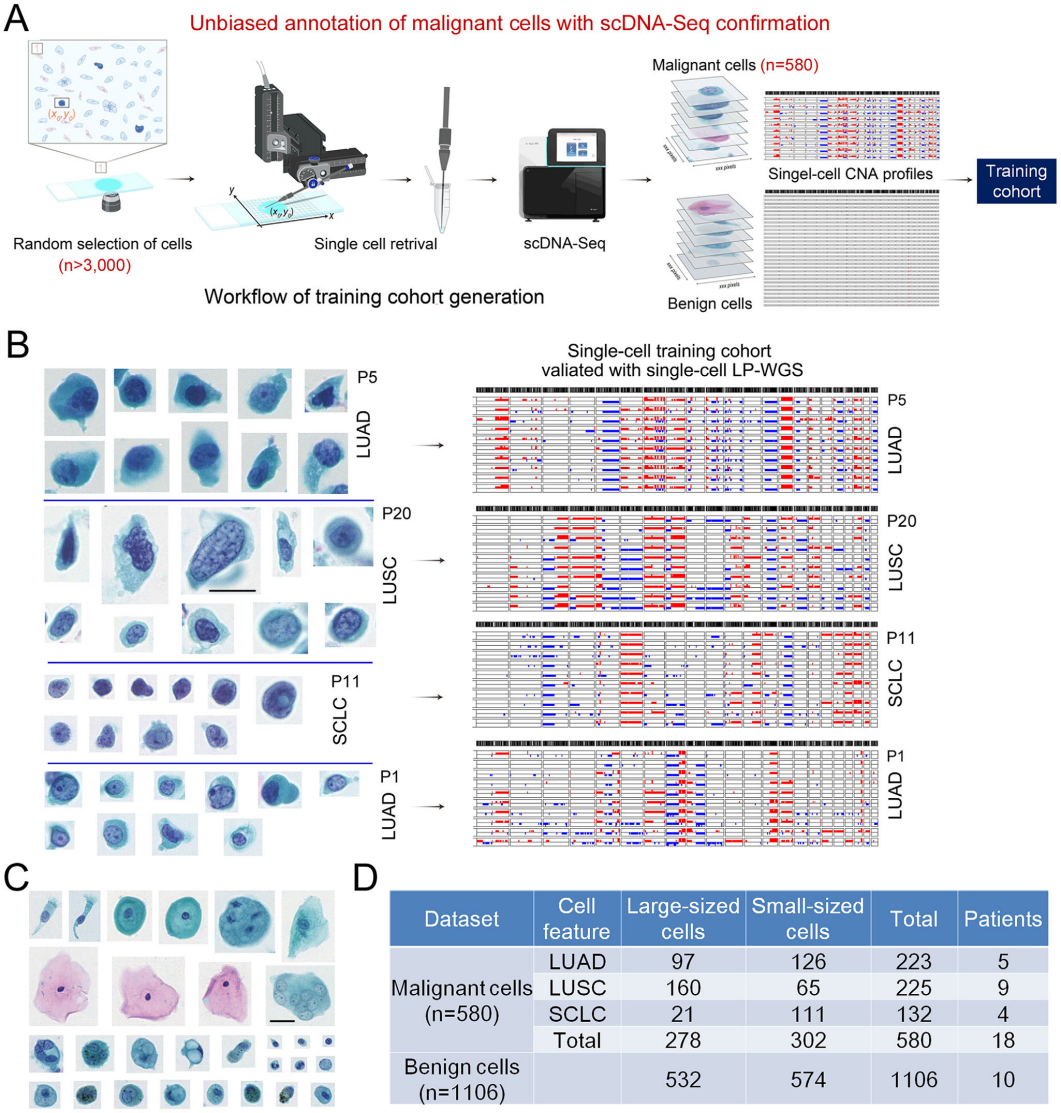
ScDNA‐Seq‐confirmed BAL ETC dataset. A) Schematic illustration of unbiased annotation of ETCs from BALF specimens with scDNA‐Seq confirmation. B) Representative Pap‐stained cytology images (left) and single‐cell CNA profiles (right) of scDNA‐Seq‐confirmed ETCs from BALF specimens of lung cancer patients. Scale bar: 20 µm. C) Representative Pap‐stained cytology images of benign cells from BALF specimens of patients with benign diseases. Scale bar: 20 µm. D) Single‐cell dataset of scDNA‐Seq‐confirmed malignant cells and benign cells for developing LESSEL. LUAD, lung adenocarcinoma; LUSC, lung squamous cell carcinoma; SCLC, small‐cell lung cancer.

A well‐annotated ETC dataset is the vital prerequisite to develop DL models for cytopathologic diagnosis of lung cancer. For unbiased and accurate annotation of ETCs, cells on the PAP‐stained BAL‐cytology slides were randomly selected and retrieved using an automated micromanipulator for scDNA‐Seq, regardless of their morphological features and biomarkers (**Figure** **2**A). In our previous studies, we developed a Tn5 transposase‐based method for rapid, low‐cost single‐cell LP‐WGS.^[^ ^20^, ^21^ ^]^

We performed Tn5‐based scDNA‐Seq on ≈3000 randomly selected cells from BALF samples of 24 lung cancer patients (Table S4, Supporting Information). A total of 580 cells were confirmed as malignant for establishing the BAL‐derived ETC dataset (Figures S4 and S5, Supporting Information). Figure 2B shows representative single‐cell CNA profiles and images of scDNA‐Seq‐confirmed ETCs from LUAD, LUSC, and SCLC patients, comprising ETCs with a spectrum of sizes and cytological features. We compared single‐cell CNA profiles of tumor cells from BAL‐cytology slides and fine needle aspiration cytology (FNAC) slides of the same patient (Figure S6, Supporting Information), and the concordance of CNA profiles clearly confirmed the tumor origin of BAL ETCs identified with scDNA‐Seq. Meanwhile, a total of 1106 benign cells, selected from 10 BALF samples of patients with benign pulmonary diseases by a senior cytopathologist (Figure 2C; Table S4, Supporting Information), were enrolled for establishing the BAL‐derived benign cell dataset. The characteristics of scDNA‐Seq‐confirmed ETC and benign cell datasets are summarized in Figure 2D.

### 2.3 Overview of LESSEL Pipeline

**Figure 3:**
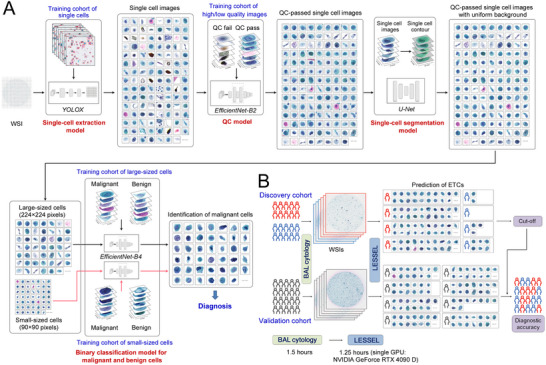
LESSEL pipeline and LESSEL‐derived diagnostic model. A) Overview of the BAL cytology‐based DL pipeline, LEESEL, for detecting ETCs in BALF specimens. B) LESSEL‐derived, WSI‐level diagnostic model for lung cancer diagnosis.

LESSEL is a DL pipeline for rapidly detecting ETCs from Pap‐stained BAL‐cytology slides at the single‐cell level (**Figure** **3**A). LESSEL first extracts single cells from a whole slide image (WSI) of the Pap‐stained BAL‐cytology slide. The raw single‐cell images are subjected to a quality control (QC) model and a single‐cell segmentation model for obtaining high‐quality single‐cell images with uniform background. The major innovation of LESSEL is the use of scDNA‐Seq for accurate, unbiased annotation of ETCs in BALF, instead of subjective annotation by cytopathologists. The scDNA‐Seq‐confirmed single‐cell dataset is used to train binary classification models for malignant and benign cells, leading to rapid ETC detection in BALF specimens. Due to high heterogeneity in cell sizes, dual‐channel classification models are generated for large‐sized and small‐sized cells, respectively. Since a WSI usually contains tens of thousands of cells, all LESSEL‐based single‐cell detections are aggregated to a primary diagnosis at the WSI‐level (Figure 3B). Model details and performance assessment are described as follows.

### 2.4 Image Preprocessing and Single‐Cell Segmentation

**Figure 4:**
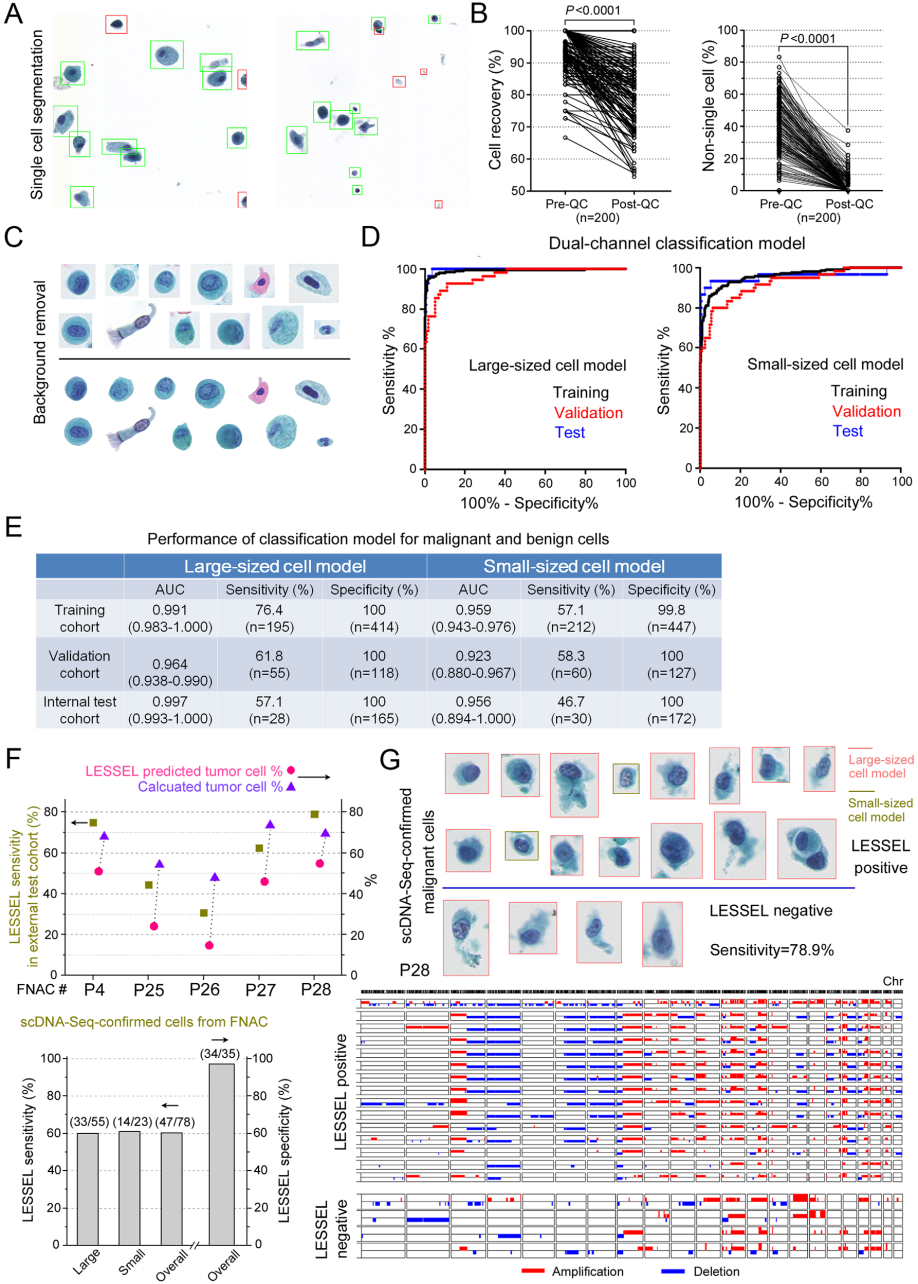
LESSEL performance. A) Single‐cell segmentation and QC model for segmenting single cells (green box) from WSIs and removing non‐single cell objects (red box). B) Left, cell recovery of the single‐cell segmentation before and after processing with the QC model (n = 200). Right, percentages of non‐single cell objects before and after the QC procedure (n = 200). The Wilcoxon signed‐rank test was performed to compare paired measurements. C) Background removal of segmented single cells (Top, before removal; Bottom, after removal). D) Receiver operating characteristic (ROC) curves of large‐sized (left) and small‐sized (right) cell classification models for malignant and benign cells. Black, training set; Red, validation set; Blue, internal test set. E) Performance of large‐sized and small‐sized cell classification models for malignant and benign cells. F) Top, LESSEL sensitivity in 78 scDNA‐Seq‐confirmed malignant cells from Pap‐stained FNAC‐cytology slides (external test set), and percentages of LESSEL predicted and calculated tumor cells in the FNAC‐cytology slides from 5 lung cancer patients. Bottom, LESSEL sensitivity and specificity in detecting malignant cells from Pap‐stained FNAC‐cytology slides in the external test set. G) Pap‐stained FNAC‐cytology images (top) and single‐cell CNA profiles (bottom) of scDNA‐Seq‐confirmed malignant cells, as well as results of LESSEL prediction. AUC, area under the ROC curve; FNAC, fine needle aspiration cytology; Chr, chromosome.

Starting with the WSI of a Pap stained‐BALF specimen, the collected WSI was cropped into non‐overlapping small images of 1024 × 1024 pixels. A YOLOX‐based single‐cell extraction model was trained (Figure S7, Supporting Information) and used to extract single‐cell images from patch‐level images, followed by an EfficientNet‐B2‐based QC model for removing non‐cellular constituents such as nuclei, debris, and incomplete cells. The performance of single‐cell extraction and QC was assessed by investigating 200 randomly selected image patches (1024 × 1024 pixels) from 10 independent WSIs. The single‐cell extraction model reported a 94.3 ± 6.9% (mean±SD, the same below) cell recovery from WSIs (**Figure** **4**A,B), but 37.7 ± 18.6% of extracted cells were found not to be intact single cells. The QC model significantly decreased the non‐single cell proportion to 3.1 ± 5.8% and exhibited 84.2 ± 11.5% cell recovery from WSIs, indicating high efficiency and accuracy of single‐cell extraction. To segment cells from the background in the single‐cell images, a U‐Net model was trained (Figure S8, Supporting Information) and employed to effectively reduce background interference and obtain single‐cell images with uniform background (Figure 4C).

### 2.5 Dual‐Channel Classification Model for Malignant and Benign Cells

All QC‐passed and segmented single cells were classified into two groups based on their cell sizes (large‐sized cells: 254 × 254 pixels; small‐sized cells: 90 × 90 pixels), and then subjected to a dual‐channel classification model for detecting large‐ and small‐sized cell malignant cells, respectively. The single‐cell dataset for LESSEL development comprised 580 scDNA‐Seq‐confirmed BAL ETCs (large‐sized: 278; small‐sized: 302) and 1106 BAL benign cells (large‐sized: 532; small‐sized: 574) across a variety of cell types from patients with benign pulmonary diseases. The dataset was split into training, validation, and internal test sets in a ratio of 7:2:1. To accurately assess the diagnostic specificity of LESSEL, the internal test set was supplemented with additional benign cells to achieve a ratio of 7:2:3 for training, validation, and internal test sets of benign cells.

A total of 195 scDNA‐Seq‐confirmed ETCs and 414 benign cells were used to train the large‐sized cell classification model for discriminating between malignant and benign cells (Figure 4D,E). Likewise, 212 ETCs and 447 benign cells were used to train the small‐sized cell classification model. We tuned the classification models to achieve a diagnostic specificity of >99.8% of malignant cell detection. This stringent threshold was set during model training to minimize false‐positive predictions. Figure 4D shows Receiver Operating Characteristic (ROC) curves of training, validation, and internal test sets by large‐ and small‐sized cell classification models. In the internal test set, the large‐sized cell classification model exhibited 57.1% sensitivity, 100% specificity and a computed Area Under the Curve (AUC) of 0.997 (95% CI: 0.993–1.000). The small‐sized cell classification model demonstrated 46.7% sensitivity, 100% specificity, and 0.956 (95% CI: 0.894–1.000) AUC (Figure S9, Table S5, Supporting Information).

To further validate classification models and investigate their generalizability, we tested them with 5 WSIs of Pap‐stained fine‐needle aspiration (or fine‐needle biopsy) cytology (FNAC) slides prepared by membrane‐based liquid‐based preparation (LBP) method as an external test set (Note: All Pap‐stained BAL‐cytology slides in this study were prepared by sedimentation LBP method). A total of 113 cells were randomly chosen from 5 Pap‐stained FNAC slides and sequenced with single‐cell LP‐WGS. Among these cells, 78 of them were confirmed malignant based on concordant single‐cell CNA profiles, as well as the remaining 35 confirmed benign due to the absence of detectable CNAs across the genome. Among scDNA‐Seq‐confirmed tumor cells, 47 out of 78 were LESSEL positive (Figure 4F), leading to 60.3% sensitivity and the averaged patient‐level sensitivity of 58.3 ± 20.4% (mean ± SD, Table S6, Supporting Information). For scDNA‐Seq‐confirmed benign cells, 34 out of 35 were tested negative with LESSEL and achieved 97.1% specificity. Figure 4G shows images and single‐cell CNA profiles of 19 scDNA‐Seq‐confirmed FNAC‐derived tumor cells from P28, including 15 LESSEL positive and 4 LESSEL negative cells. In the WSI of P28, 1985 cells were found LESSEL positive in a total of 3622 cells, representing 54.8% of tumor cell proportion in FNAC. Considering the 79.0% LESSEL sensitivity in tumor cell detection, the accurate tumor cell proportion was calculated to be 69.4% (Figure 4F), similar to the tumor cell proportion calculated based on scDNA‐Seq results (69.0%, 78 out of 113). Overall, this FNAC‐derived external test set validated the generalizability of LESSEL.

### 2.6 Clinical Investigation of LESSLE for BAL‐Based Cytopathologic Diagnosis of Lung Cancer

**Figure 5:**
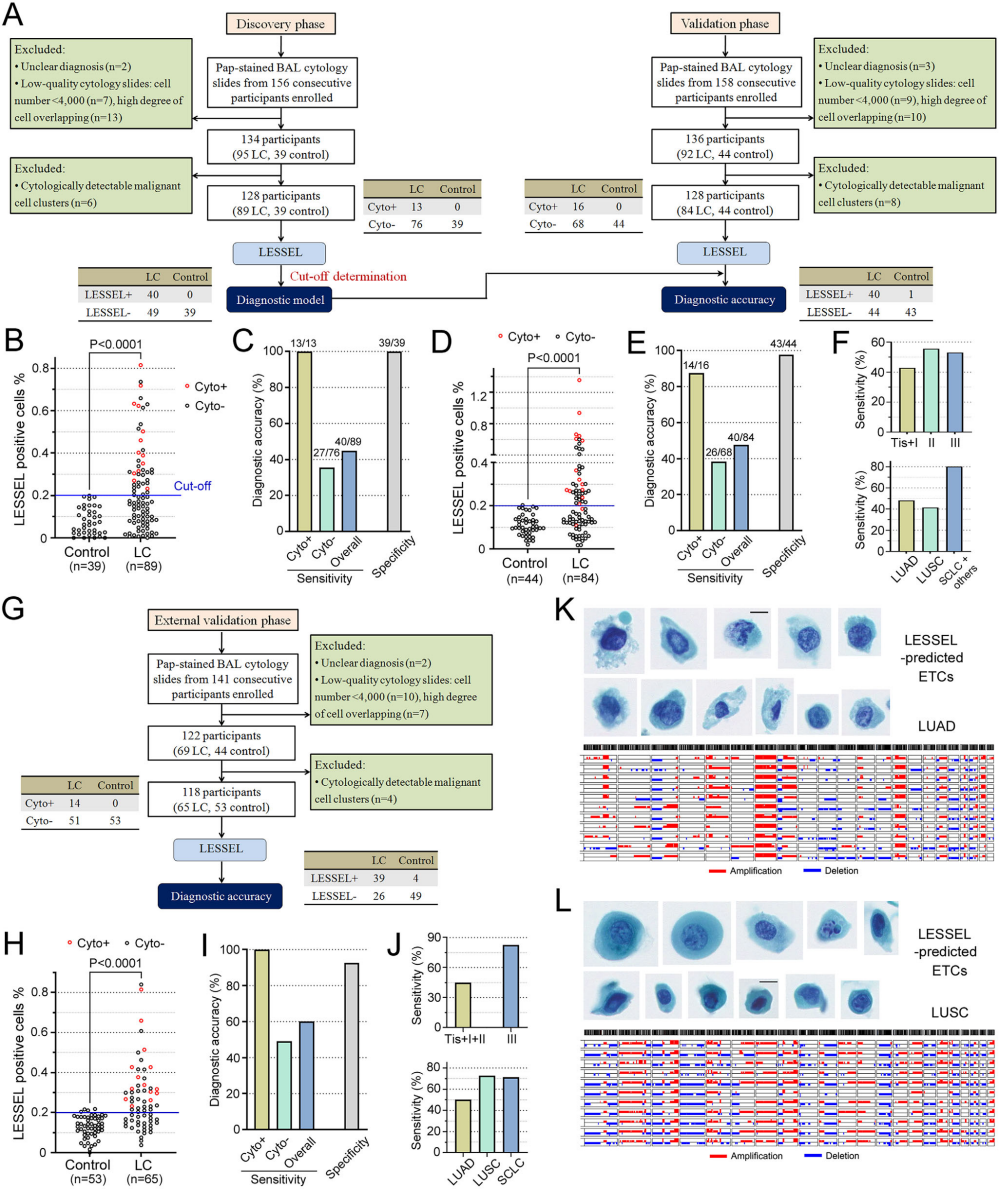
Clinical investigation of LESSEL for BAL‐based cytopathologic diagnosis of lung cancer. A) Flowchart of development and validation of LESSEL‐derived diagnostic model and the eligible participants, and the description of discovery and validation cohorts (center 1, see Methods). B) LESSEL‐predicted BAL ETC% in clinically diagnosed lung cancer (n = 89) and control (n = 39) groups from the discovery cohort for identifying optimal cut‐off. C) Diagnostic performance of LESSEL‐derived diagnostic model in diagnosing BALF specimens from the discovery cohort. D) LESSEL‐predicted BAL ETC% in the validation cohort including lung cancer (n = 84) and control (n = 44) groups. E) Diagnostic performance of LESSEL‐derived model in diagnosing BALF specimens from the validation cohort. F) Diagnostic sensitivities of the LESSEL‐derived model in diagnosing lung cancer with different stages and histology subtypes. G) Description of external validation cohort (center 2, see Methods). H) LESSEL‐predicted BAL ETC% in the external validation cohort including lung cancer (n = 65) and control (n = 53) groups. I) Diagnostic performance of LESSEL‐derived model in diagnosing BALF specimens from the external validation cohort. J) Diagnostic sensitivities of the LESSEL‐derived model in diagnosing lung cancer with different stages and histology subtypes. Single‐cell CNA profiles of LESSEL‐predicted ETCs from a stage III, LUAD patient (K, patient #19) and a stage II, LUSC patient (L, patient #15) in the external validation cohort. The Mann–Whitney test was performed to compare LC and control groups. Cyto, cytology; LC, lung cancer; LUAD, lung adenocarcinoma; LUSC, lung squamous cell carcinoma; SCLC, small‐cell lung cancer.

LESSLE detects ETCs from WSIs of Pap‐stained BAL‐cytology slides at the single‐cell level. Since BAL‐cytology slides usually contain tens of thousands of cells, the output of LESSEL serves as input for a WSI‐level model of lung cancer diagnosis. To develop this WSI‐level diagnostic model, WSIs from a retrospective cohort of 156 consecutive patients who underwent BAL were collected and analyzed with LESSEL as a discovery phase. A flow diagram summarizing the eligible participants is shown in **Figure** **5**A, and detailed demographics and clinical characteristics of participants are shown in **Table** **1**. Twenty‐two participants were excluded due to unclear diagnosis or low‐quality cytology slides, as well as 6 participants excluded by cytopathologists because of cytologically detectable ETC clusters. WSIs of Pap‐stained BAL cytology from 128 patients (89 lung cancer, 39 control) were then analyzed with LESSEL (Table S7, Supporting Information). Representative LESSEL‐predicted ETCs were shown in Figures S10 and S11 (Supporting Information). An optimal cut‐off of LESSEL‐predicted ETC % (the number of LESSEL‐predicted ETCs/number of total cells) was determined at 0.2% (Figure 5B). At this cut‐off, the diagnostic model detected all (13/13, 100%) cytology‐positive BALF specimens and 27 of 76 (35.5%) cytology‐negative BALF specimens, producing 207% more positive findings than BAL cytology. Overall, the diagnostic model showed 44.9% sensitivity, 100% specificity, 100% PPV, and 44.3% NPV in diagnosing BALF specimens (Figure 5C), significantly outperforming BAL cytology with improved sensitivity (44.9% vs 14.6%; P = 0.0001) and same specificity (100% vs 100%).

**Table 1:** Patient characteristics and LESSEL‐predicted BAL ETC% in the discovery and validation cohorts.

		Discovery Cohort (n = 156)			Validation Cohort (n = 158)			External Validation (n = 141)		
		LC	Control	*P* ^a)^	LC	Control	*P* ^a)^	LC	Control	*P* ^a)^
Participant included for analysis		89	39		84	44		65	53	
Age (years)	Mean (SD)	60 (11)	58 (13)	0.36	62 (10)	57 (16)	0.11	62 (11)	56 (15)	0.02
Sex	female	47/42	17/22	0.34^b)^	48/36	23/21	0.60 ^b)^	46/19	27/26	0.03^b)^
Tumor subtype	LUAD	61 (69%)			50 (59%)			36 (55%)		
	LUSC	17 (19%)			29 (35%)			22 (34%)		
	SCLC	8 (9%)			2 (2%)			7 (11%)		
	Others	3 (3%)			3 (4%)			0 (0%)		
Tumor stage	Tis+I	61 (69%)			48 (58%)			37 (57%)		
	II	13 (15%)			18 (21%)			5 (8%)		
	III	15 (16%)			18 (21%)			23 (35%)		
Benign diseases	Infection		21			23			27	
	BPN		13			14			23	
	Tuberculosis		4			3			3	
	Others		1			4			0	
ETC %, median (IQR)		0.173 (0.083–0.310)	0.067 (0.024–0.131)	<0.0001	0.179 (0.120‐0.296)	0.104 (0.086–0.139)	<0.0001	0.232 (0.156–0.319)	0.133 (0.095–0.176)	<0.0001

^a)^ P value for comparing the characteristics between LC and control groups. Mann‐Whitney test was performed for comparison;

^b)^ Pearson's chi‐square test was used for comparison. SD, standard deviation; Tis, Tumor in situ; BPN, Benign pulmonary nodule; IQR, interquartile range.

To validate the LESSEL‐derived diagnostic model, we compared its diagnostic performance with BAL cytology in an independent cohort. As shown in Figure 5A, 158 consecutive patients who underwent BAL were enrolled, and 22 participants were excluded due to unclear diagnosis or low‐quality cytology slides, as well as 8 participants excluded because of cytologically detectable ETC clusters. The LESSEL‐derived model diagnosed BALF specimens with a 47.6% sensitivity, 97.7% specificity, 97.6% PPV, and 49.4% NPV (Figure 5D), significantly surpassing BAL cytology with improved sensitivity (47.6% vs 19.0%; *P* < 0.0001) and comparable specificity (97.7% vs 100%). In particular, the diagnostic model detected 14 of 16 (87.5%) cytology‐positive BALF specimens and 26 of 68 (38.2%) cytology‐negative BALF specimens from lung cancer patients. The diagnostic method detected 46.3% (31/67) early lung cancer (stage Tis, I, II) and 52.9% (9/17) locally advanced lung cancer (stage III). To validate the LESSEL‐derived diagnosis of cytology‐negative BALF specimens, scDNA‐Seq was used to investigate the malignancy of LESSEL‐predicted ETCs. In a stage I LUAD patient, LESSEL detected 152 ETCs from the Pap‐stained BAL cytology slide, which had been classified as cytology‐negative (Figure S12, Supporting Information). We retrieve 28 LESSEL‐predicted ETCs for scDNA‐Seq, and 18 of them (64.3%) exhibited concordant CNA profiles (Figure S13, Supporting Information), confirming their malignancy and establishing the malignant diagnosis of this BALF specimen.

To assess the generalizability of the diagnostic model, we further investigate it using an external validation cohort from an independent center (n = 141, Figure 5G). The LESSEL‐derived model diagnosed BALF specimens with a 60.0% sensitivity, 92.5% specificity, 90.7% PPV, and 65.3% NPV (Figure 5H), significantly outperforming BAL cytology with improved sensitivity (60.0% vs 21.5%, *P* < 0.0001) while maintaining comparable specificity (92.5% vs 100%, P = 0.12). The diagnostic model detected all (14/14) cytology‐positive BALF specimens and 25 of 51 (49.0%) cytology‐negative BALF specimens from lung cancer patients (Figure 5I). Additionally, it detected 45.2% (19/42) early lung cancer and 82.6% (19/23) locally advanced lung cancer (Figure 5J). We performed scDNA‐Seq to validate the malignancy of LESSEL‐predicted ETCs in cytology‐negative samples. In a stage III LUAD patient, LESSEL detected 38 ETCs in the BALF sample, and 16 ETCs were collected for scDNA‐Seq, with 11 exhibiting concordant CNA profiles (Figure 5K). Likewise, in a stage II LUSC patient, LESSEL detected 45 ETCs from the BALF sample, and among the 14 retrieved for scDNA‐Seq, 11 exhibited concordant CNA profiles (Figure 5L). These scDNA‐Seq results confirmed the malignancy of LESSEL‐predicted ETCs and established definitive lung cancer diagnoses. Compared with the validation cohort, enhanced sensitivity in the external validation cohort can be attributed to a higher proportion of stage III lung cancer patients (35.4% vs 20.2%).

## 3 Discussion

To develop robust and effective DL models, a high‐quality annotated dataset is regarded as a vital prerequisite. In conventional AI‐based cytology, experienced cytopathologists identify different types of cells based on their cytological features as the ground truth or reference for model training and test‐validation. Large‐scale manual annotation and engineering of feature extraction are time‐consuming and require advanced skills of cytopathologists. Due to substantial overlapping cytologic features between benign cells and ETCs, inaccurate and incomplete annotation of ETCs is common in exfoliative cytology, especially in BAL cytology that suffers from low sensitivity in diagnosing malignant lung lesions. Thus, large‐scale, accurate annotation of BAL ETCs is technically difficult and demanding for cytopathologists. Moreover, China had only 20400 registered pathologists in 2022, despite an estimated demand of nearly 100000.^[^ ^30^ ^]^ Cytopathologists make up only ≈10% of pathologists in major hospitals and face an overwhelming workload, limiting their capacity for detailed data annotation.

To address this challenge, the major innovation of this study is the use of scDNA‐Seq as an objective method for accurate, unbiased ETC annotation from BALF specimens, instead of subjective annotation by cytopathologists. Single‐cell LP‐WGS characterizes genome‐wide CNA profiles that create an objective ground truth for malignant cell identification. The rapid, low‐cost Tn5‐based scDNA‐Seq method enables single‐cell LP‐WGS of thousands of cells randomly selected from BALF specimens in a few weeks. In this study, ≈3000 cells from Pap‐stained BAL‐cytology slides of 24 lung cancer patients were randomly chosen and individually sequenced, and a total of 580 cells were confirmed as ETCs with a variety of morphologies and subtypes of lung cancer. Unlike subjective identification of cytologic morphologies, scDNA‐Seq represents an objective criterion to accurately annotate BAL ETCs and provides a standard and scalable means for ETC annotation in different types of body fluids. The integration of automated robotics for single‐cell and liquid manipulation has the potential to significantly accelerate this technique.

We chose BAL cytology to test the scDNA‐Seq‐guided strategy for training DL models because BAL is a commonly used procedure for early lung cancer diagnosis. The majority of lung cancer cases are diagnosed in an advanced stage, significantly impacting survival rates. The 5‐year survival rate declines from 60% for stage IIA to 36% for stage IIIA and further drops to 6% for stage IV.^[^ ^22^, ^23^ ^]^ Although pathologic evaluation of tissue biopsy remains the gold standard for cancer diagnosis, biopsy is an invasive procedure with attendant morbidity and rare mortality. In lung cancer, percutaneous transthoracic needle biopsy (PTNB) has a diagnostic sensitivity of 92.5%, but it carries notable complications including pneumothorax (22.7%) and hemorrhage (7.1%).^[^ ^31^ ^]^ Electromagnetic navigation bronchoscopy (ENB) offers a 79.8% sensitivity with pneumothorax (5.2%) being its most common complication.^[^ ^31^ ^]^ Compared with invasive biopsy, BAL is a safe, easily performed, minimally invasive, and well‐tolerated procedure. BALF samples are collected and transported to the laboratory within 2 h. BAL cytology is then performed and takes ≈1.5 h. However, BAL cytology suffers from low sensitivity for diagnosing malignant lung lesions (≈14.7%),^[^ ^26^, ^27^ ^]^ especially in BALF specimens that are free of cytologically detectable ETC clusters. In this study, we have developed LESSEL, a BAL cytology‐based DL pipeline, for cytopathological diagnosis of lung cancer by detecting ETCs in BALF specimens. The LESSEL‐derived diagnostic model significantly outperformed BAL cytology with improved diagnostic sensitivity and comparable diagnostic specificity. In particular, the diagnostic model detected ≈46% early lung cancer (stage Tis, I and II), significantly surpassing BAL cytology. In addition, LESSEL, as a deep learning model, analyzes WSIs of Pap‐stained BAL cytology slides without requiring any modifications in sample processing or cytology testing. It functions as an automated end‐to‐end pipeline, providing both images of LESSEL‐predicted ETCs and diagnostic results for BALF samples. Its seamless integration into clinical workflows eliminates the need for re‐training cytopathologists, making it easily adaptable in clinical practice. LESSEL is rapid and cost‐effective, superior to costly and time‐consuming molecular tests such as cfDNA analysis, which would largely reduce the burden on patients.

LESSEL has three major advantages over other cytology‐based DL models. First, LESSEL is highly scalable for large training datasets through large‐scale scDNA‐Seq, which can be performed by technicians rather than senior cytopathologists. By employing a random cell collection strategy combined with scDNA‐Seq, this approach minimizes bias and variability from cytopathologists, resulting in a more objective and unbiased dataset for training DL models. As the size of the scDNA‐Seq‐confirmed dataset increases, LESSEL's performance improves, enabling it to detect subtle morphological features that may not be readily apparent to human observers. Second, LESSEL represents a general approach for developing cytology‐based DL models and could be easily transferred to other types of body fluids (e.g., urine, pleural effusion, ascites, bile, and blood). Third, LESSEL detects ETCs in BALF at the single‐cell level, making it more explainable than patch‐based DL models in literature, and its prediction of ETCs can be further validated with scDNA‐Seq for assessing the model performance with single‐cell precision.

A notable limitation of this study is the relatively small size of the scDNA‐Seq‐confirmed dataset that resulted in relatively suboptimal sensitivity in diagnosing malignant BALF specimens, especially those from LUAD patients. The low proportion of ETCs in LUAD BALF specimens caused low efficiency in scDNA‐Seq‐based ETC identification, and thereby a relatively small size of the scDNA‐Seq‐confirmed LUAD cell dataset. However, the dataset is scalable and can be expanded by incorporating additional scDNA‐Seq‐confirmed ETCs. In this study, the clinical utility of LESSEL is still preliminary as the validation cohort size is small, thereby warranting a large‐scale prospective clinical trial. Another apparent shortcoming of LESSEL, as a single‐cell DL model, is its inability to detect ETC clusters due to their sizes ranging from a few cells to hundreds of cells and their presence only in a small fraction of BALF specimens. Meanwhile, cytopathologists accurately identify ETC clusters in BAL fluids–demonstrated by our single‐cluster sequencing results (Figure 1C: sensitivity 87.2%, specificity 88.2%, PPV 89.5%, NPV 85.7%). Given this expertise, AI assistance is unnecessary to cytopathologists when ETC clusters are present in the BALF specimens. However, the real challenge for cytopathologists lies in accurately diagnosing BALF specimens that lack detectable ETC clusters. Thus, we developed LESSEL for rapid and accurate detection of single ETCs in BALF specimens while excluding samples with cytologically detectable ETC clusters from clinical cohorts. Third, the robustness (different sample collection, preparation and staining protocols, equipment) of LESSEL warrants large‐scale, multi‐center validation in an independent cohort. In addition, the generalizability of LESSEL requires further validation. We tested LESSEL with different LBP methods (sedimentation and membrane‐based) and Pap‐stained FNAC slides. However, LESSEL still needs to be tested with different staining protocols and image quality variation caused by different scanners. Fourth, LESSEL detects ETCs from Pap‐stained BAL cytology slides to aid in cancer diagnosis; however, it is unable to distinguish lung cancer patients by stage or subtype.

## 4 Conclusion

A DL approach for BAL‐based cytopathologic diagnosis of lung cancer has been developed based on an accurate and unbiased BAL ETC dataset using scDNA‐Seq as an objective ground truth for ETC annotation. This scDNA‐Seq‐guided ETC annotation strategy offers an efficient solution to generate an objective and accurately annotated ETC dataset for training DL models, significantly reducing reliance on expert annotations. As a cost‐effective method, LESSEL has the potential to improve diagnosis of early lung cancer and could be adapted to other types of body fluids for cytopathologic diagnosis of malignancy, leading to a significant advancement in liquid biopsy.

## 5 Experimental Section

### Study Design and Cohort Description

The main objective was to develop LESSEL, a DL pipeline, for detecting ETCs in BALF, and to explore the utility of LESSEL for cytopathologic diagnosis of lung cancer. The study was performed at The First Affiliated Hospital, Zhejiang University School of Medicine (Hangzhou, China) from January 2024 to March 2025. All samples were anonymously coded and technicians were blinded to the clinical information. All images were obtained from archival glass slides of Pap‐stained BAL cytology specimens. This study involved four cohorts: 1) a DL pipeline training and validation cohort consisting of 34 patients (24 lung cancer patients and 10 control subjects) for generating Pap‐stained, single‐cell image dataset; 2) a discovery cohort for generating LESSEL‐derived diagnostic model of 156 consecutive patients who received routine bronchoscopy from the First Affiliated Hospital, Qingchun campus (Hangzhou, 310003, China, center 1); 3) a validation cohort of 158 consecutive patients who received routine bronchoscopy from center 1 for assessing diagnostic accuracy; and 4) an external validation cohort of 141 consecutive patients who received routine bronchoscopy from the First Affiliated Hospital, Yuhang campus (Hangzhou, 311121, China, center 2). The discovery, validation, and external validation cohorts were retrospectively enrolled from the first week of January 2024, the second week of January 2024, and the last two weeks of May 2024, respectively. All clinical diagnoses of BALF specimens were based on the following criteria. The malignant BALF diagnoses were confirmed by surgical or biopsy histopathology. In this study, the biopsy was performed after BAL during routine bronchoscopy, and surgery was performed within one week of BAL. The benign BALF diagnosis was defined as i) at least 1 negative biopsy histopathology or cytologic evaluation, ii) the patient had no evidence of malignancy at 6 months follow‐up; and iii) a strong alternative etiology that was suspected or diagnosed. Subjects with suspected lung cancer, who were finally diagnosed with another respiratory‐related benign disease (including pneumonia, bronchitis, sarcoidosis, and chronic bronchopathy) were classified as controls. Sex has been carefully considered as a biological variable in this investigation. Samples from males and females were tested with the information presented in Supporting Tables.

### Dataset Description

The Pap‐stained, single‐cell image dataset involved 580 scDNA‐Seq‐confirmed ETCs from BALF specimens of 24 lung cancer patients and 1106 benign cells from BALF specimens of 10 patients with benign pulmonary diseases (see Data, Materials and Software Availability).

### BALF Collection

BALF samples were collected during bronchoscopy for routine diagnostic purposes. An electronic bronchoscope was inserted through the nasal cavity and flashed the airways and the lesion site determined by imaging techniques with saline fluid to harvest surrounding cells. Lavage was performed using 100 mL of 37 °C normal saline in 5 fractions through a disposable spray catheter, followed by collection of the fluid into a sterile collection bottle containing 10 mL of liquid‐based cell preservation solution (AccuPath, Guangzhou, China) by 0.01 MPa negative pressure suction. Approximately 20–50 mL BALF was obtained and transported to the laboratory within two hours at room temperature. BALF was centrifuged at 1200–1500 rcf for 15 min at 4 °C and the cell‐free supernatant decanted off from the cell pellet.

### BAL Cytology

The cells from BALF were sedimented onto the adhesive slides and then subjected to Pap staining using a high‐throughput LBP‐2264 cell sedimentation & slide stainer integrated workstation (AccuPath, Guangzhou, China), followed by coverslipping with a Leica CV5030 robotic coverslipper (Leica Biosystems, Nussloch, Germany). The Pap‐stained BAL‐cytology slides were scanned into WSIs using a KF‐PRO‐400‐HI slide scanner (KFBIO, Yuyao, China) for digital analysis.

### Unbiased Annotation of ETCs by Random Cell Selection and Sequencing

A senior cytopathologist selected Pap‐stained BAL cytology slides from 24 lung cancer patients (Table S4, Supporting Information), covering different stages and subtypes, and randomly selected areas on each slide for single cell collection. In the selected areas, all single cells were retrieved with a micromanipulator (EppendrofTransferMan 4r) for scDNA‐Seq. This strategy enables rapid and random collection of single cells for sequencing, leading to unbiased ETC annotation.

### Tn5 Transposome Assembly

Commercial Tn5 transposase was purchased from Novoprotein (China). Transposon DNA oligonucleotides were synthesized by Genewiz (China) and diluted with an annealing buffer to a concentration of 100 mм. To form Tn5 transposome, Tn5‐ME (CTGTCTCTTATACACATCT, 10 µL) and Tn5‐adaptor1 (TCGTCGGCAGCGTCAGATGTGTATAAGAGACAG, 10 µL) or Tn5‐adaptor2 (GTCTCGTGGGCTCGGAGATGTGTATAAGAGACAG,10 µL) oligonucleotides were mixed together at an equimolar ratio, and annealed by gradual cooling (75 °C 15 min, 60 °C 10 min, 50 °C 10 min, 40 °C 10 min, and 25 °C 30 min). The preannealed transposon oligonucleotides mixture (4 µL) was subsequently mixed with Tn5 transposase (20 µL, 10 mм), followed by incubation for 1 h at room temperature. The assembled Tn5 transposome was stored at −20 °C.

### Low‐Cost Single‐Cell WGS Using Tn5 Transposome

For single‐cell WGS, a cell was collected by a micromanipulator into a low‐binding PCR tube (200 µL, Axygen) containing 4.0 µL of cell lysis buffer (10 mм Tris‐EDTA pH 8.3, 0.3% Triton X‐100, 20 mм NaCl, 0.5 μм carrier ssDNA, 1 mg mL^−1^ QIAGEN protease, 15 mм DTT). The PCR tube was incubated at 55 °C for 3 h to lyse the cell and release genomic DNA, followed by denaturing protease at 80 °C for 1 h. Exposed genomic DNA was tagmented by adding 0.5 µL of Tn5 transposome (1:200 diluation, 5× reaction buffer: 50 mм TAPS‐NaOH, 25 mм MgCl2, 40% PEG 8000, pH 8.5) that introduced PCR adaptor to DNA fragments. 0.1% SDS was added and incubated at 55 °C for 1 h to stop the tagmentation and expose DNA, followed by adding triton X‐100 at a final concentration of 0.1% for neutralizing SDS. After DNA fragmentation, gap filling of fragmented genomic DNA was conducted at 72 °C for 10 min with NEBNext Ultra II Q5 Master Mix (M0544, New England Biolabs), followed by polymerase‐based fragmented DNA and library amplification. PCR condition was 98 °C for 1 min, 20 cycles of 98 °C for 10 s, 60 °C for 15 s, and 72 °C for 30 s. Amplified single‐cell sequencing library was purified with Agencourt AMPure XP beads. High‐throughput sequencing was conducted on NovaSeq 6000 (PE150).

### Bioinformatics Analysis

A list of bioinformatic analysis tools used in this study were summarized in the following Table with detailed parameters available on request. For CNA analysis, FASTQ files were aligned to the major chromosomes of human (hg19) using BWA (version 0.7.17) with default options. PCR duplicates were removed with Samtools (version 1.11). Aligned reads were counted in fixed bins averaging 500 kb. Bin counts were normalized for GC content with low regression and bin‐wise ratios were calculated by computing the ratio of bin counts to the sample mean bin count. The diploid regions were determined using HMMcopy (version 0.1.1). Segmentation was performed with the circular binary segmentation (CBS) method (alpha = 0.0001 and undo.prune = 0.05) from R Bioconductor ʻDNAcopyʼ package. Copy number noise was quantitated using the mean absolute pairwise difference (MAPD) algorithm. Samples with MAPD≤ 0.45 passed the MAPD QC and were included in single‐cell CNA analyses.

### CNA Burden Calculation and scDNA‐Seq‐Bsed ETC Identification

CNA burden was defined as the percentage of the tumor autosomal genome with copy number altered. To calculate CNA burden for a sample, segments of copy number gains and losses were determined (see Code Availability for codes), and their total genomic length was summed and calculated as a percentage of the size of the autosomal genome. Multiple cells with detectable copy number alterations (CNAs) exhibit concordant single‐cell CNA profiles that were confirmed as ETCs based on a quantitative criterion developed in the previous study.^[^ ^19^ ^]^

### Image Preprocessing

The collected WSIs were split into non‐overlapping small images of 1024 × 1024 pixels. In this study, the OpenCV (version 4.9.0) python package was used to process and crop slide‐level WSIs into patch‐level images of 1024 × 1024 pixels in ʻPNGʼ format.

### Single‐Cell Extraction Model

A YOLOX network was trained to extract single‐cell images from patch‐level images. The network was trained on 219 patch‐level images, split into training and validation sets in a 9:1 ratio.

(1) $$L_{\mathrm{total}}=\lambda_{\mathrm{reg}}L_{\mathrm{IoU}}+L_{\mathrm{obj}}+L_{\mathrm{cls}}$$

(2) $$L_{\mathrm{IoU}}=\;\frac{1}{n_{\mathrm{fg}}}\sum_{i\;=\;1}^{n_{\mathrm{fg}}}\left(1-{\left(\frac{\left|b_{\mathrm{fg}}^{\left(i\right)}\cap b_{\mathrm{gt}}^{\left(i\right)}\right|}{\left|b_{\mathrm{fg}}^{\left(i\right)}\cup b_{\mathrm{gt}}^{\left(i\right)}\right|}\right)}^{2}\right)\;$$

The total loss was a weighted sum of the intersection over union (IoU) loss (L_IoU_), objectness loss (L_obj_), and classification loss (L_cls_) (Equation (1)). The foreground sample number (n_fg_), the area of the predicted box (b_fg_), and the area of the real box (b_gt_) were utilized to calculate the IoU loss (Equation (2)). The ʻ*BCEWithLogitsLossʼ* function was adopted from PyTorch (version 2.2.1) to calculate both objectness loss and classification loss. During the training process, 0.5 was used as the regularization weight (λ_reg_) for the IoU loss. The batch size was set to 64 and the model parameters were updated using the SGD optimizer with Nesterov Accelerated Gradient (momentum factor = 0.9) and L2 weight decay was set to 5 × 10^−4^. The ʻ*yoloxwarmcosʼ* learning rate scheduling strategy was adopted from the YOLOX open‐source framework. A warm‐up phase of 5 epochs followed by cosine annealing was combined. During the warm‐up phase, the learning rate increased from 0 to 0.01 first, then gradually decreased to 5 × 10^−4^ and remained at the same level. During the inference phase, the network took patch‐level images as input and output annotations in ʻjsonʼ format. The detected regions were retained with a confidence score greater than 0.3 and an IoU value greater than 0.5 after applying Non‐Maximum Suppression (NMS). The foreground bounding boxes of these regions were then cropped and used as input for the next stage.

### Quality Control (QC) Model

After single‐cell extraction, a QC model was employed to remove non‐cellular constituents such as nuclei, debris, and incomplete cells. A dataset was generated of low‐quality images, comprising 6 categories including blurry cells, incomplete cells, cell fragments, multicellular clumps, impurities, and cell nuclei. The extracted single‐cell images were resized to a uniform size of 224 × 224 pixels, and processed with a seven‐class classification model that was trained from a pre‐trained EfficientNet‐B2 network for filtering out low‐quality images. The fully connected layer of the original EfficientNet‐B2 network was replaced with a sequential module. The module consisted of a linear layer that mapped the input features to the desired number of classes, followed by a softmax activation function applied along the specified dimension. Under the supervision of 757 high‐quality single‐cell images and 2071 low‐quality images, split into training and validation sets in a 7:3 ratio, the model was fine‐tuned using an SGD optimizer with an L2 weight decay of 4 × 10^−4^ and a momentum of 0.96. The learning rate scheduling strategy involved a linear increase from 9 × 10^−5^ to 4 × 10^−4^, followed by a sustain phase over five epochs, and finally an exponential decay with a decay factor of 0.93. The batch sizes of 8 and the epochs of 50 were adapted to suit the datasets. The high‐quality single‐cell images obtained from this stage were used for subsequent downstream analysis.

### Single Cell Segmentation Model

A U‐Net model was trained to segment cells from the background in the single‐cell images to effectively reduce background interference. A total of 1423 single‐cell images were annotated using AnyLabeling (version 0.3.3) to generate labeled ʻjsonʼ files, which were then converted into mask images in ʻpngʼ format. These images were split into training and validation sets in a 9:1 ratio. The classic U‐Net network, consisting of 19 CNN blocks, was trained on these cell and mask images for 20 epochs. The RMSprop optimizer alongside the ʻ*ReduceLROnPlateau*ʼ learning rate scheduling strategy was employed from PyTorch (version 2.2.1) to optimize the Dice score. A patience value of 7 was set for the scheduler, with an initial learning rate of 2 × 10^−5^. Additionally, a weight decay of 1 × 10^−8^, a momentum of 0.95, and batch sizes of 4 to update the model parameters were utilized. The overall loss function combined the ʻ*BCEWithLogitsLoss*ʼ from PyTorch and the Dice loss. This combination ensured comprehensive learning by assessing both pixel‐wise accuracy and region‐based similarity between the predicted segmentation and the ground truth masks. During the inference phase, the input images were resized to 224 × 224 pixels, and only the predicted regions containing single cells were retained for further analysis.

### Dual‐Channel Classification Model for Malignant and Benign Cells

Cells from BALF specimens were highly heterogeneous in cell sizes. For this reason, cells were categorized into large‐sized and small‐sized cell groups (large‐sized cells: 254 × 254 pixels; small‐sized cells: 90 × 90 pixels) to develop two separate binary classification models. These results showed enhanced performance of two classification models than a single classification model consisting of cells with different sizes. In the preparation stage, the input size for the large‐sized cell classification model was set at 224 × 224 pixels, while the classification model for small‐sized cells used 90 × 90 pixels as the input size. The single‐cell dataset comprised a total of 580 scDNA‐Seq‐confirmed BALF‐derived ETCs (large‐sized: 278; small‐sized: 302) and 1106 BALF‐derived benign cells (large‐sized: 532; small‐sized: 574) across a variety of cell types from patients with benign pulmonary diseases. The dataset was split into training, validation, and test sets in a ratio of 7:2:1. To accurately assess the specificity of the models, the test sets were supplemented with additional benign cells to achieve a ratio of 7:2:3 for training, validation, and internal test sets of benign cells. Two pre‐trained EfficientNet‐B4 networks were employed with similar modifications to the previously described QC model. Specifically, the original fully connected layer was replaced in both networks with a sequential module. This module included a linear layer mapping input features to two output classes, followed by a softmax activation function. The learning rate scheduling strategy and optimizer settings for both EfficientNet‐B4‐based models were aligned with those of the QC model. Two models were optimized separately, using identical batch sizes of 8 and a consistent training duration of 70 epochs to ensure model convergence.

### Statistical Analysis

Data were reported as means with standard deviations (SDs) and median with inter‐quartile range (IQR), as appropriate. Statistical significance was considered when the two‐tailed P value was less than 0.05 for all statistical tests. The normality of the data was tested by the Kolmogorov‐Smirnov test. The Mann–Whitney test was performed for the non‐parametric test between two groups that were not normally distributed. The Wilcoxon signed‐rank test was performed to compare paired measurements. The receiver operating characteristic (ROC) curve was generated to compute the area under the curve (AUC) with a 95% Wald confidence interval (CI). Sensitivity and specificity were calculated using stand 2 × 2 contingency tables. The Pearson's chi‐square test was performed on contingency tables. Statistical analyses were performed with GraphPad Prism 9.

### Ethics

All patient samples were obtained from participants who provided informed consent in accordance with the revised Declaration of Helsinki Guidelines (2013) and with approval from the institutional review board of The First Affiliated Hospital, Zhejiang University School of Medicine (#20240212). All research on human participants was performed in accordance with the approved protocols.

## Conflict of Interest

A patent has been filed in China by Fudan Zhang Jiang Institute and Fudan University.

## Supporting information



Supporting Information

Supplemental Table 5

Supplemental Table 6

## Data Availability

The data that support the findings of this study are openly available in Genome Sequence Archive under reference number HRA008613.
