# Supplementary material for: Single‐Cell Sequencing‐Guided Annotation of Rare Tumor Cells for Deep Learning‐Based Cytopathologic Diagnosis of Early Lung Cancer
Source: Adv Sci (Weinh). 2025 Apr 15;12(22):2416921. doi: 10.1002/advs.202416921 (PMC12165082; doi:10.1002/advs.202416921)
Supplement: Supplementary file 1 — Supporting Information [file ADVS-12-2416921-s003.pdf]

ADVANCED SCIENCE
Open Access

Supporting Information

for *Adv. Sci.*, DOI 10.1002/advs.202416921

Single-Cell Sequencing-Guided Annotation of Rare Tumor Cells for Deep Learning-Based Cytopathologic Diagnosis of Early Lung Cancer

Yichun Zhao, Ruoran Qiu, Zhuo Wang, Yunyun Li, Xu Yang, Yanlin Li, Xiaohan Shen, Yun Liu, Ziqiang Chen, Qihan You* and Qihui Shi**

Supporting Information

Single-cell sequencing-guided annotation of rare tumor cells for deep learning-based cytopathologic diagnosis of early lung cancer

Yichun Zhao, Ruoran Qiu, Zhuo Wang, Yunyun Li, Xu Yang, Yanlin Li, Xiaohan Shen, Yun Liu, Ziqiang Chen, Qihan You,* Qihui Shi**

Supplementary Methods

Reagents and software

Reagent	Source	Identifier
Triton™ X-100	Sigma-Aldrich	#X100
SDS,10%	Solarbio	#S1010
QIAGEN Protease	QIAGEN	#19157
NaCl (5 M)	Invitrogen	#AM9760G
$MgCl_2$ (1 M)	Invitrogen	#AM9530G
NaOH (10 M)	Sigma-Aldrich	#72068
Tris-EDTA Buffer 100x concentrate	Sigma-Aldrich	#T9285
DTT	Sigma-Aldrich	#D0632
PEG8000	Sangon	#A600433
TAPS	Sigma-Aldrich	#T5130
NovoNGS® Tn5 Transposase	Novoprotein	#M045-01A
NEBNext® Ultra™ II Q5® Master Mix	New England Biolabs	#M0544
Qubit dsDNA HS Assay Kit	Invitrogen	#Q32854
Agencourt® AMPure XP beads	Beckman Coulter	#A63881
UltraPure™ DNase/RNase-Free Distilled Water	Invitrogen	#10977015

Software & algorithms	Source	Website
FastQC (v0.11.9)	N/A	https://www.bioinformatics.babraham.ac.uk/projects/fastqc/
Trimmomatic (v0.39)	Bolger et al., 2014, 10.1093/bioinformatics/btu170	https://www.bioinformatics.babraham.ac.uk/projects/fastqc/
Cutadapt (v4.6)	Martin 2011, 10.14806/ej.17.1.200	https://cutadapt.readthedocs.io/en/stable/
BWA (v0.7.17)	Li and Durbin 2010, 10.1093/bioinformatics/btp698	http://bio-bwa.sourceforge.net
Samtools (v1.11)	Heng et al., 2009, 10.1093/bioinformatics/btp352	https://samtools.sourceforge.net/

HMMcopy (v1.32.0)	Lai et al., 2018, HMMcopy: Copy number prediction with correction for GC and mappability bias for HTS data.	https://rdrr.io/bioc/HMMcopy/ https://github.com/oicr-gsi/hmmcopy
Annovar (202200823)	Wang et al., 2010, 10.1093/nar/gkq603	http://annovar.openbioinformatics.org/en/latest/
Sentieon (202108)	Freed et al., 2017, 10.1101/115717	https://support.sentieon.com/appnotes/# https://github.com/Sentieon/sentieon-dnaseq
GraphPad Prism	N/A	RRID:SCR_002798
YOLOX	Ge et al., 2021, YOLOX: Exceeding YOLO series in 2021. arXiv:2107.08430*.	https://github.com/Megvii-BaseDetection/YOLOX
Unet	Ronneberger et al., 2015, U-Net: Convolutional Networks for Biomedical Image Segmentation. arXiv:1505.04597*.	https://github.com/milesial/Pytorch-UNet
EfficientNet	Tan and Le, 2021, EfficientNetV2: Smaller Models and Faster Training. arXiv:2104.00298*.	https://github.com/lukemelas/EfficientNet-PyTorch
R scripts for single-cell CNA analysis	This study	https://github.com/Shilab-wangzhuo/CNV
LESSEL	This study	https://github.com/Shilab-wangzhuo/LESSEL

Supplementary Figures

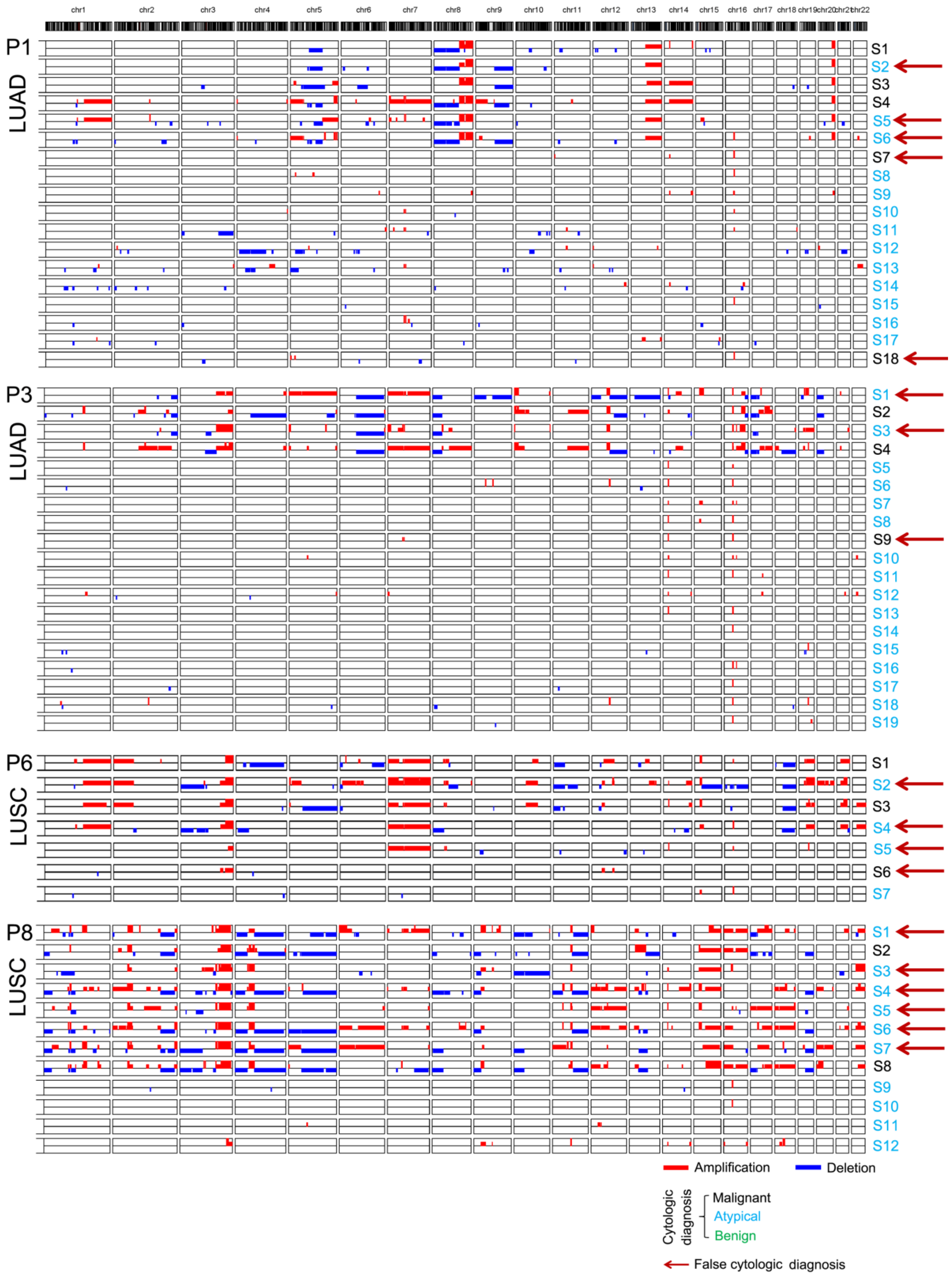

Figure S1. Single-cell CNA profiles of BALF-derived cells from P1, P3, P6 and P8 with annotation of a senior cytologist. The red arrows represent incorrect cytological evaluation based on single-cell CNA profiling results.

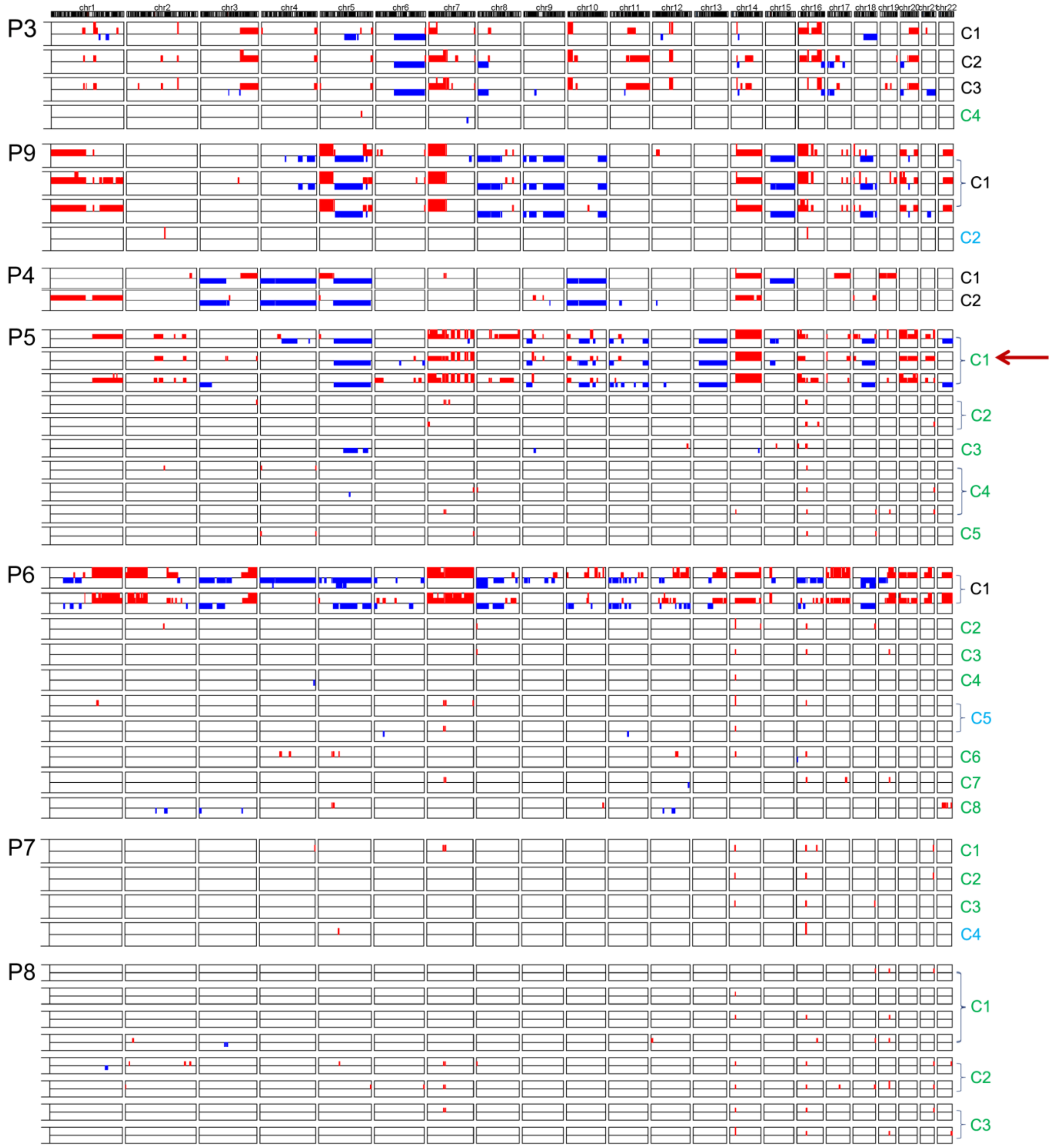

Figure S2. Single-cell CNA profiles of BALF-derived cell clusters from P3-P9 with annotation of a senior cytologist. The red arrows represent incorrect cytological evaluation based on single-cell CNA profiling results.

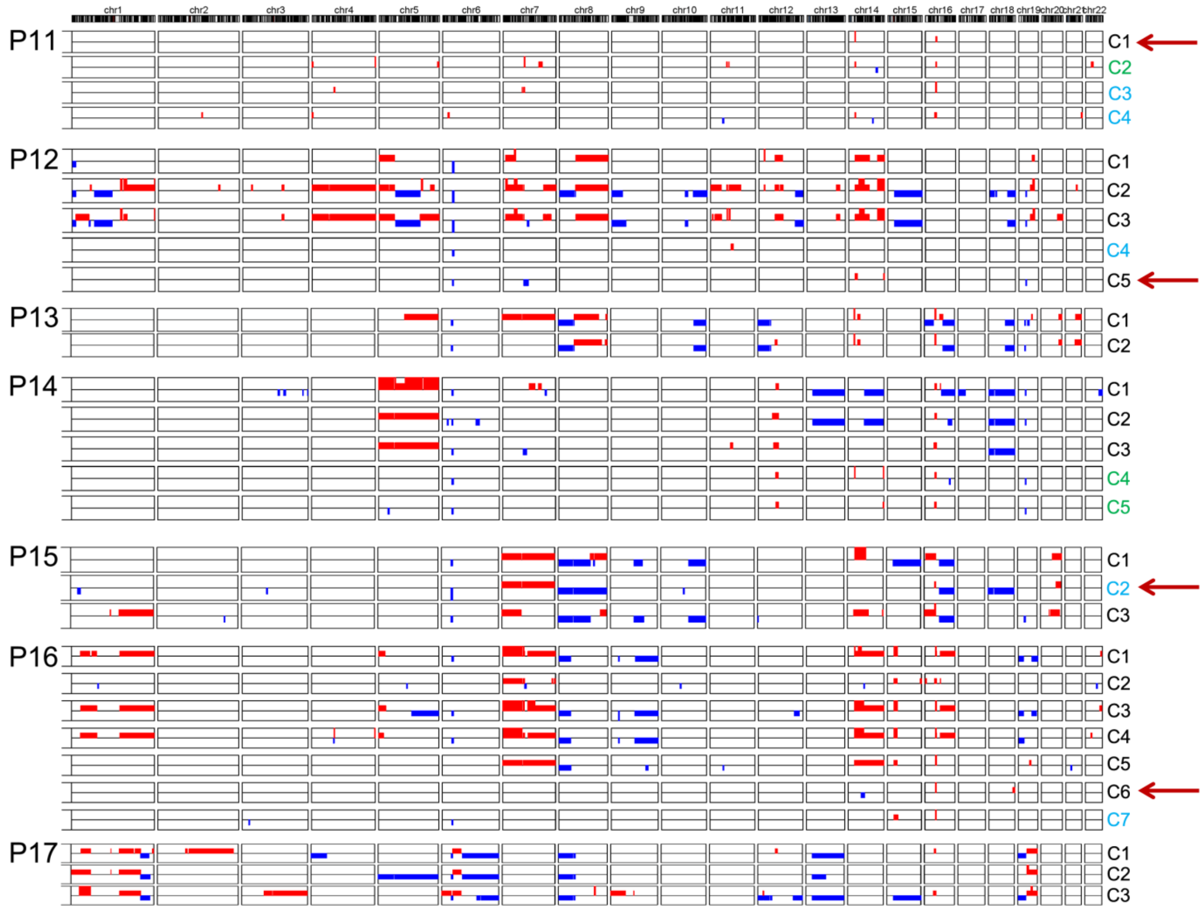

Figure S3. Single-cell CNA profiles of BALF-derived cell clusters from P11-P17 with annotation of a senior cytologist. The red arrows represent incorrect cytological evaluation based on single-cell CNA profiling results.

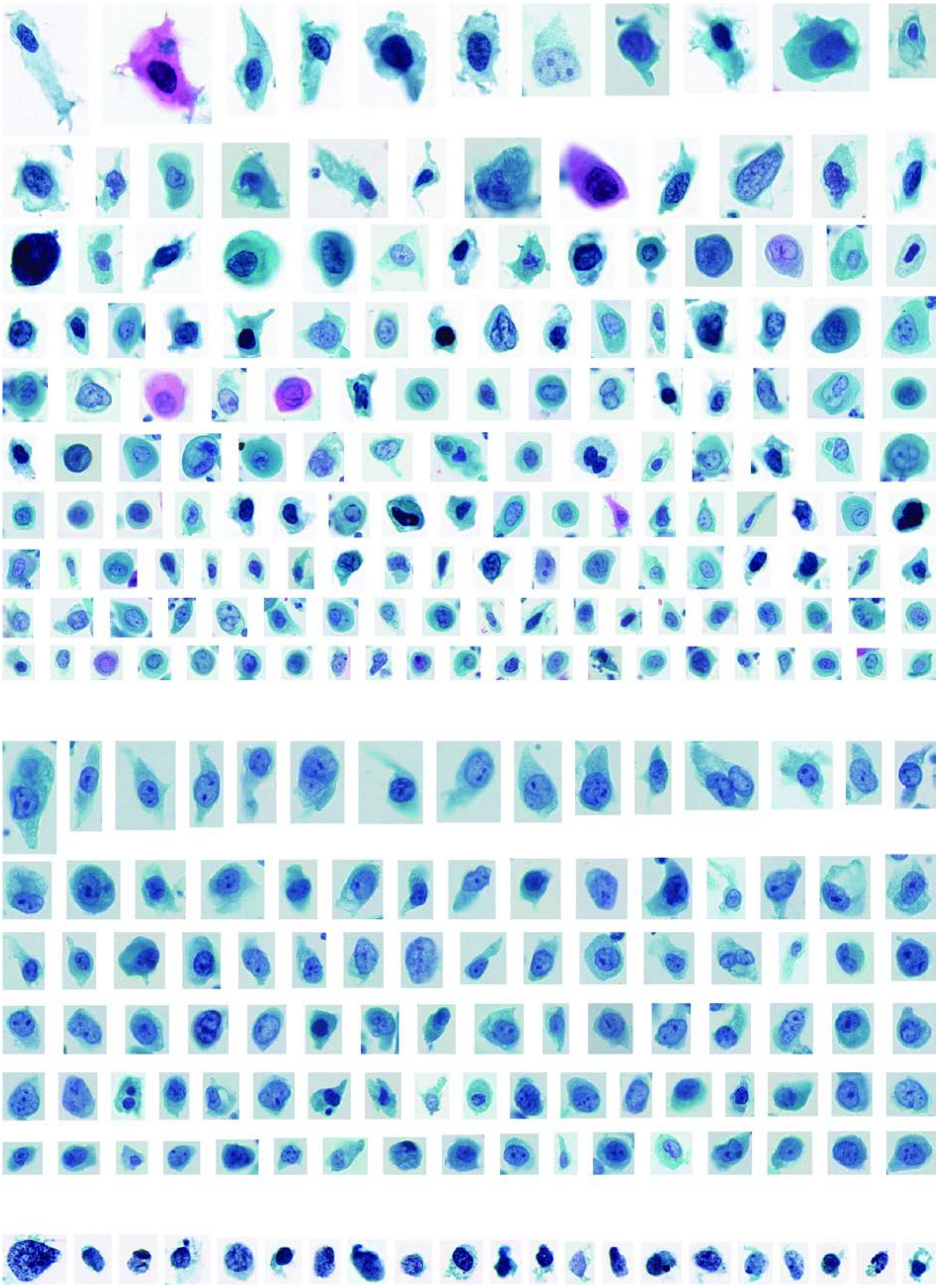

Figure S4. ScDNA-Seq-confirmed, large-sized malignant cells (n=278) from Pap-stained BAL-cytology slides. Top, LUSC (n=160); Middle, LUAD (n=97); Bottom, SCLC (n=21).

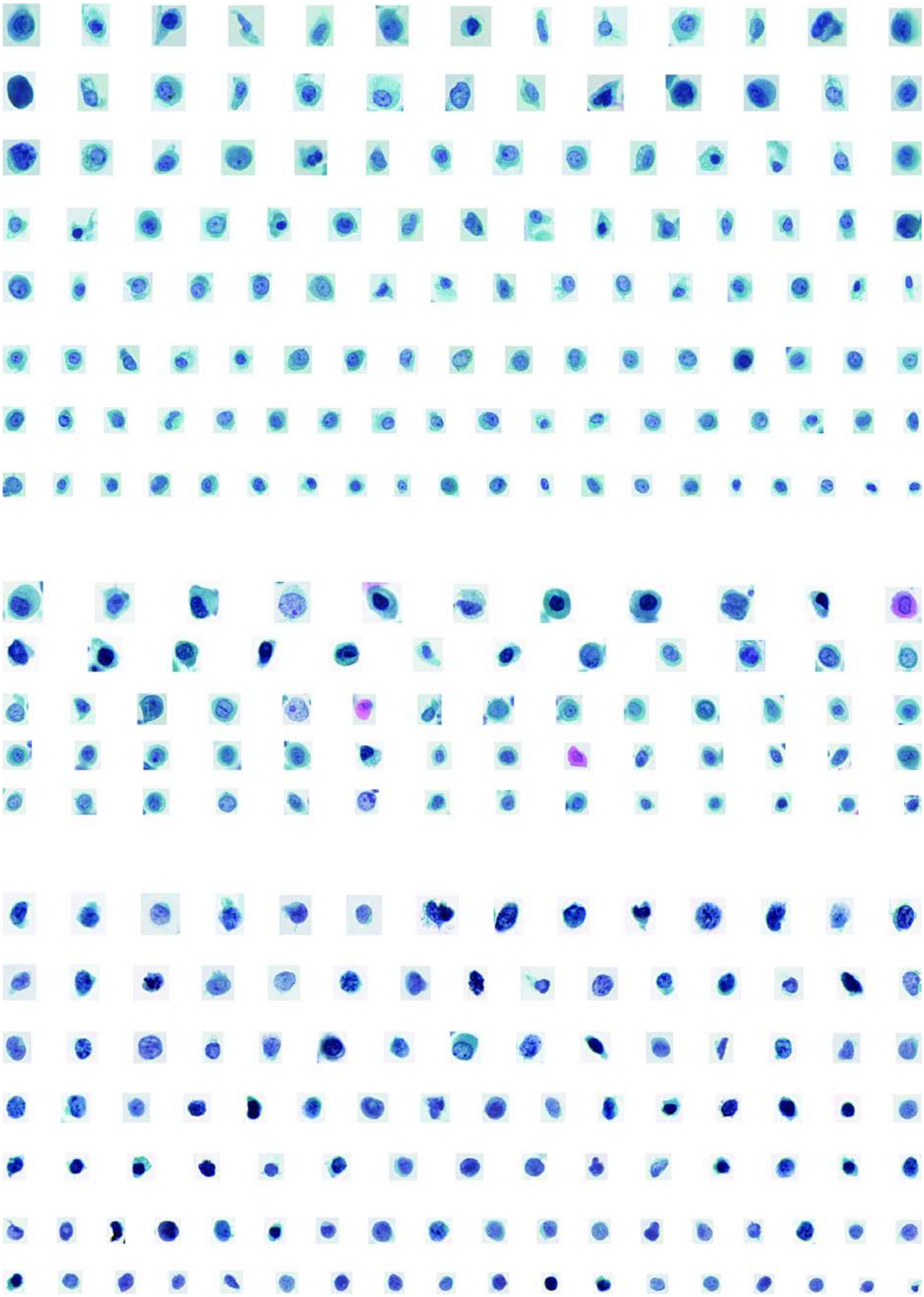

Figure S5. ScDNA-Seq-confirmed, small-sized malignant cells (n=302) from Pap-stained BAL-cytology slides. Top, LUAD (n=126); Middle, LUSC (n=65); Bottom, SCLC (n=111).

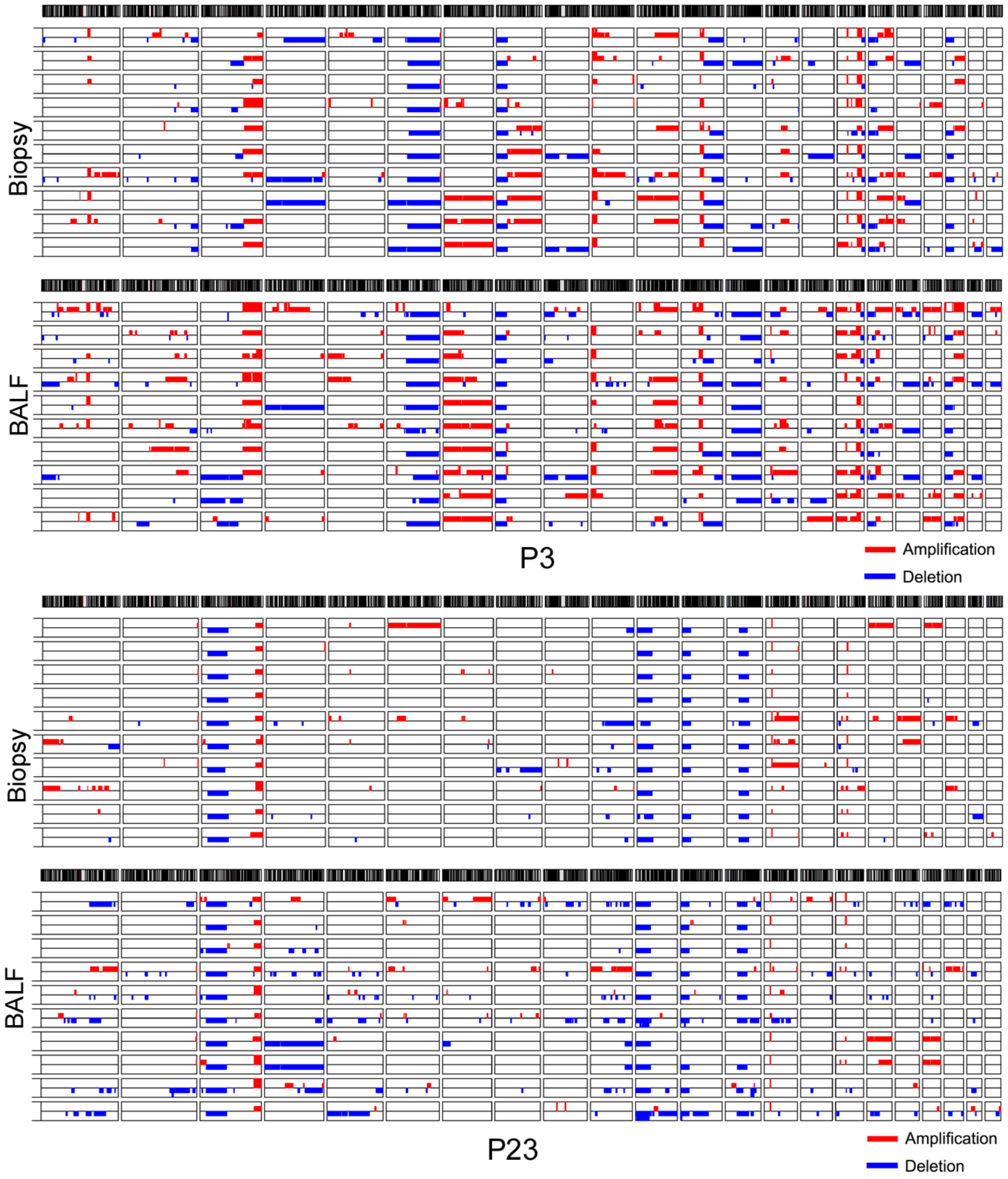

Figure S6. Single-cell CNA profiles of tumor cells from BALF samples and tissue biopsy in P3 (top, LUAD) and P23 (bottom, SCLC).

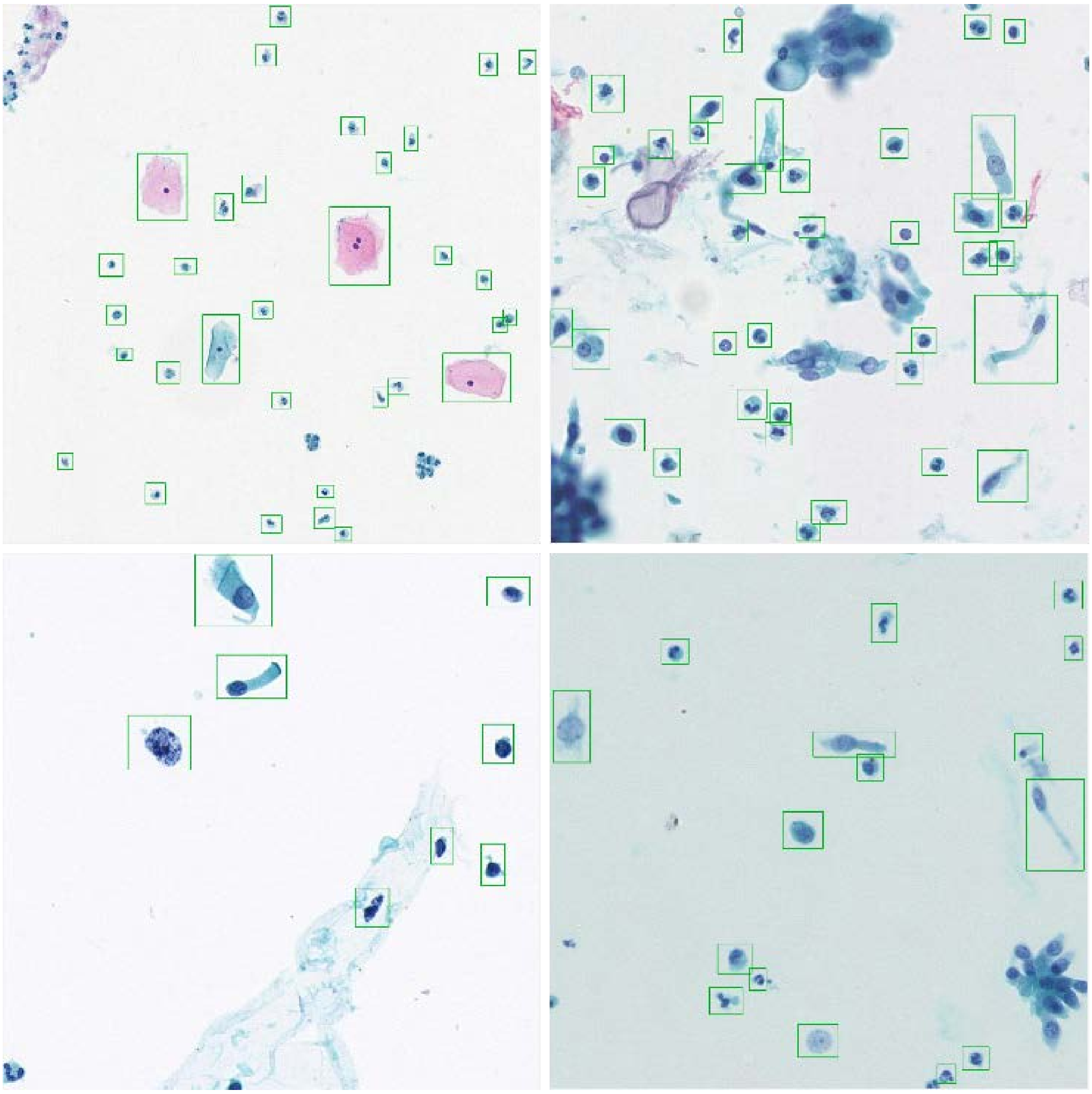

Figure S7. Reprehensive images of single cell annotation for training YOLOX model (single-cell extraction model). WSIs of 21 Pap-stained BAL cytology slides were firstly cropped into 1024×1024 pixel PNG patches. A total of 219 patches with different background and color styles were selected for annotating single cells with AnyLabeling (version 0.3.3). Single cells with clear boundaries were manually marked with green rectangular boxes. A total of 5,496 annotation boxes were created in 219 patches, which were split into a training set and a validation set in a 9:1 ratio (198:21) for training the YOLOX model.

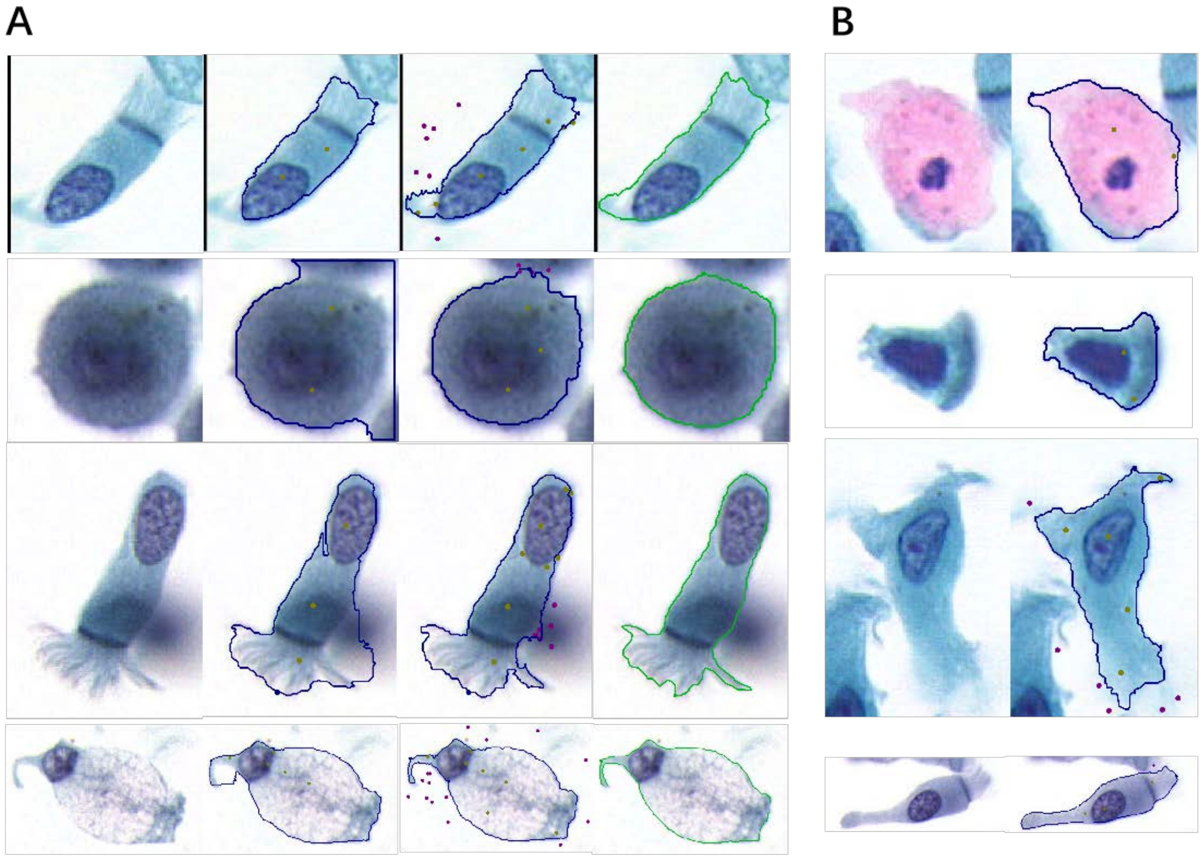

Figure S8. Reprehensive images of cell segmentation annotation for training U-Net model (single cell segmentation model). The automatic annotation feature of AnyLabeling, containing the Segment Anything model, was used to annotate masks for single cell segmentation. A total of 1,423 single cell images were annotated for generating mask files. (A) Images with low-quality performance of automatic annotation in the U-Net model training set. Single cells masks were manually corrected after automatic annotation. The blue contours were generated by automatic annotation, and the green contours denoted manually adjusted mask for U-Net model training. (B) Images with high-quality performance of automatic annotation in the U-Net model training set.

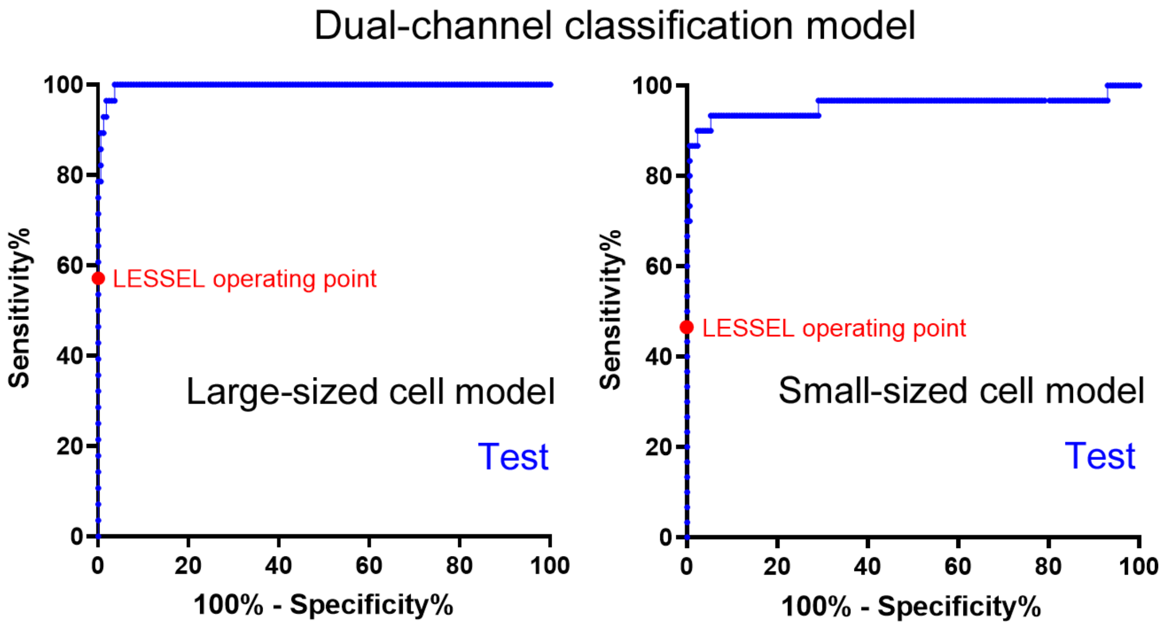

Figure S9. ROC curves of large-sized (left, AUC 0.997) and small-sized (right, AUC 0.956) cell classification models for malignant and benign cells in the internal test groups.

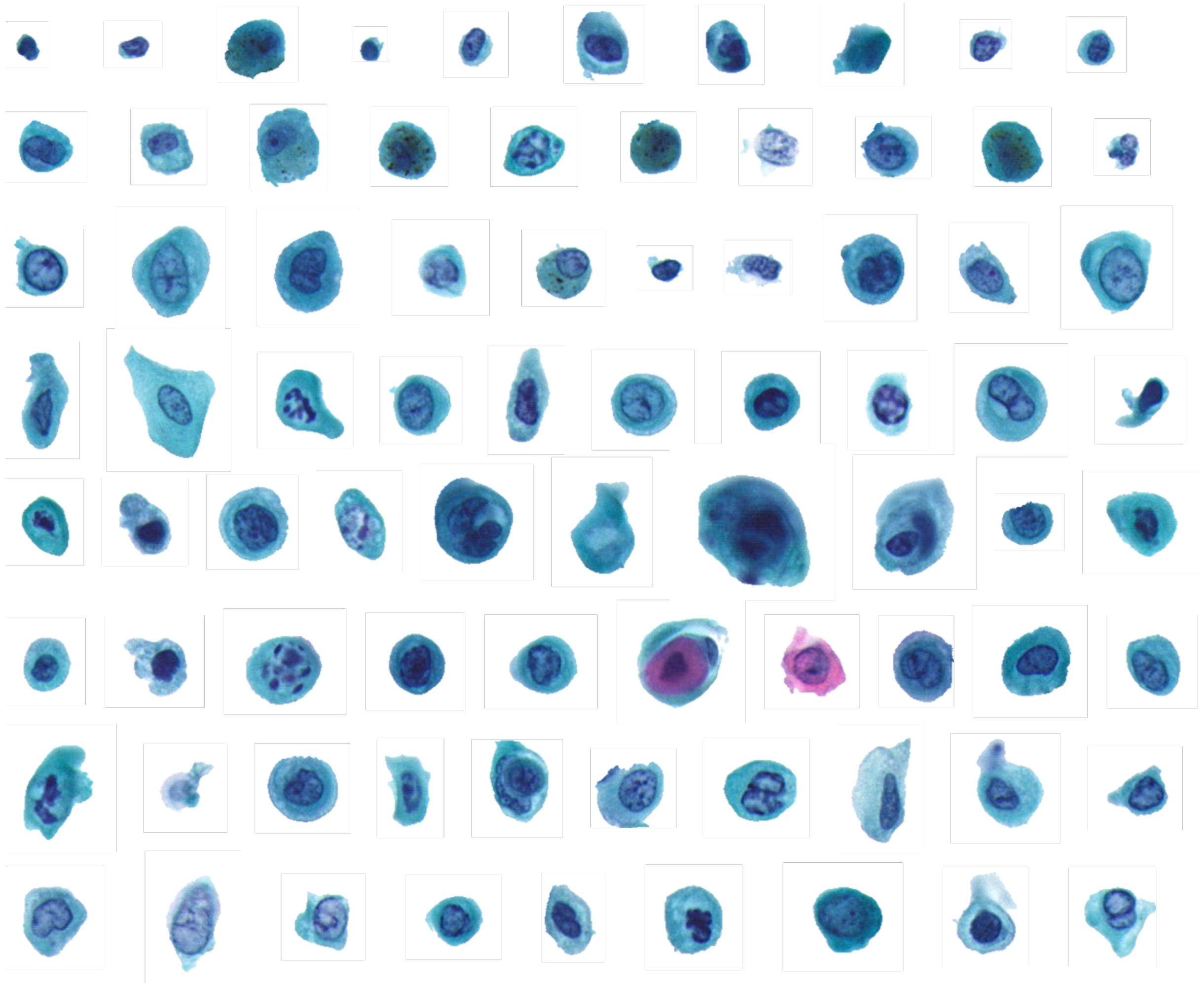

Figure S10. Pap-stained images of 79 LESSEL-predicted ETCs from lung cancer patient #4 in the discovery cohort (see: https://github.com/Shilab-wangzhuo/LESSEL for demonstration of LESSEL).

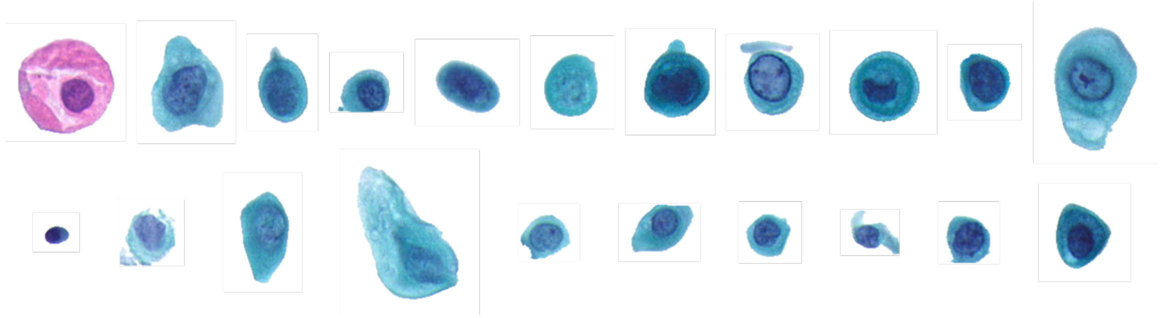

Figure S11. Pap-stained images of 21 LESSEL-predicted ETCs from lung cancer patient #9 in the discovery cohort (see: https://github.com/Shilab-wangzhuo/LESSEL for demonstration of LESSEL).

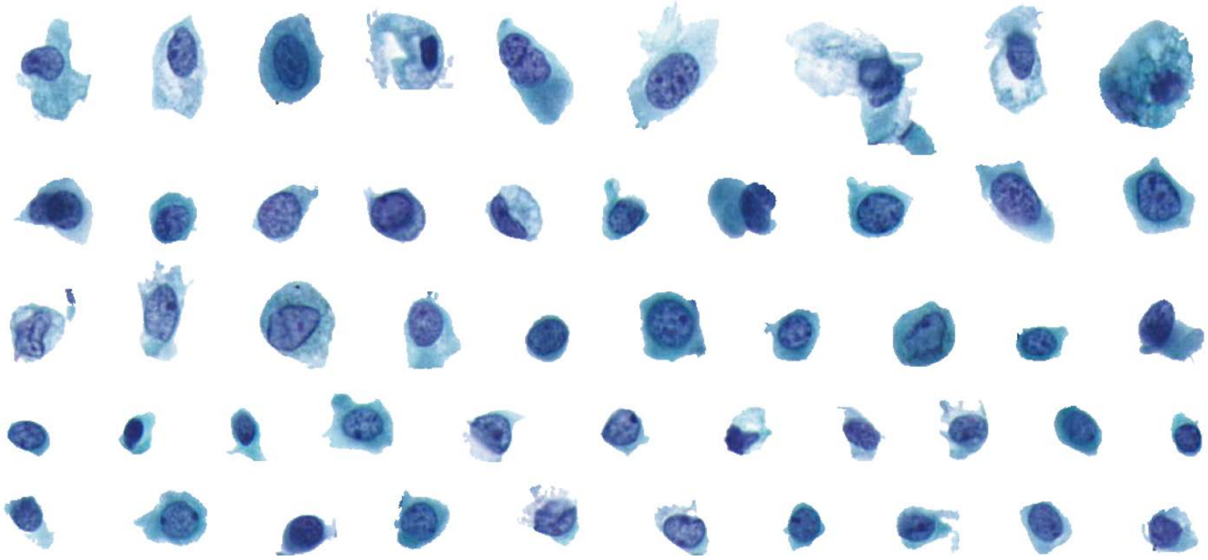

Figure S12. Pap-stained images of 50 representative LESSEL-predicted ETCs from a stage I, LUAD patient in the validation cohort (patient #35, center 1).

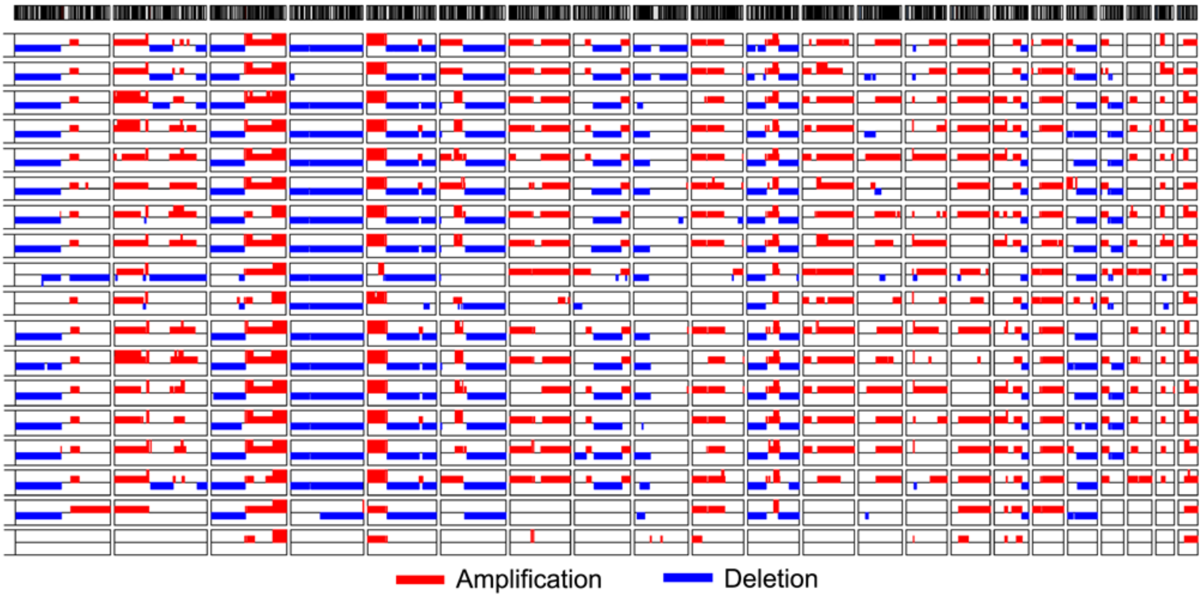

Figure S13. Single-cell CNA profiles of 18 LESSEL-predicted ETCs from a stage I, LUAD patient in validation cohort (patient #35, center 1). A total of 28 LESSEL-predicted ETCs were sequenced and 18 of them exhibited concordant CNA profiles. The remaining 10 cells were found absent of detectable CNAs.

Supplementary Tables

Table S1. Demographics and clinical characteristics of lung cancer patients that were used to investigate the consistency of BAL-derived single cell and cell cluster identification between BAL cytological evaluation and scDNA-Seq.

No	Age	Sex	Subtype	Stage
P1	75	M	LUAD	IB
P2	71	M	LUAD	IIA
P3	66	F	LUAD	IIIC
P4	75	F	SCLC	IIIA
P5	74	F	LUAD	IIIC
P6	70	M	LUSC	IIIB
P7	58	F	LUSC	IA
P8	68	M	LUSC	IIB
P9	68	M	LUAD	IVA
P10	67	M	LUSC	IIIA
P11	80	M	SCLC	N/A
P12	80	M	LUAD	IB
P13	48	F	LUAD	IIB
P14	54	F	LUAD	IVB
P15	51	M	LUAD	IIA
P16	47	F	LUAD	IVA
P17	76	F	LUAD	IVB

Table S2. Consistency of cell malignancy identification by cytology and scDNA-Seq. Of note, BAL cytology was dichotomized as diagnostic for benign if atypical or benign identified and diagnostic for malignancy if malignancy was identified. A total of 85 single cells from 8 patients (P1-P8) were scrutinized.

Patient No.	Single cell No.	Cytology	scDNA-Seq	Consistency
P1	S1	malignant	malignant	Y
	S2	atypical	malignant	N
	S3	malignant	malignant	Y
	S4	malignant	malignant	Y
	S5	atypical	malignant	N
	S6	atypical	malignant	N
	S7	malignant	benign	N
	S8	atypical	benign	Y
	S9	atypical	benign	Y
	S10	atypical	benign	Y
	S11	atypical	benign	Y
	S12	atypical	benign	Y
	S13	atypical	benign	Y
	S14	atypical	benign	Y
	S15	atypical	benign	Y
	S16	atypical	benign	Y
	S17	atypical	benign	Y
	S18	malignant	benign	N
P2	S1	malignant	malignant	Y
	S2	malignant	malignant	Y
	S3	malignant	malignant	Y
	S4	atypical	benign	Y
	S5	benign	benign	Y
	S6	malignant	benign	N
	S7	atypical	benign	Y
	S8	atypical	benign	Y
	S9	atypical	benign	Y
	S10	atypical	benign	Y
	S11	atypical	benign	Y
	S12	atypical	benign	Y
	S13	atypical	benign	Y
P3	S1	atypical	malignant	N
	S2	malignant	malignant	Y
	S3	atypical	malignant	N
	S4	malignant	malignant	Y
	S5	atypical	benign	Y
	S6	atypical	benign	Y

	S7	atypical	benign	Y
	S8	atypical	benign	Y
	S9	malignant	benign	N
	S10	atypical	benign	Y
	S11	atypical	benign	Y
	S12	atypical	benign	Y
	S13	atypical	benign	Y
	S14	atypical	benign	Y
	S15	atypical	benign	Y
	S16	atypical	benign	Y
	S17	atypical	benign	Y
	S18	atypical	benign	Y
	S19	atypical	benign	Y
P4	S1	atypical	malignant	N
	S2	atypical	malignant	N
	S3	atypical	malignant	N
	S4	atypical	benign	Y
P5	S1	malignant	malignant	Y
	S2	malignant	malignant	Y
	S3	atypical	malignant	N
	S4	malignant	malignant	Y
	S5	atypical	benign	Y
	S6	malignant	benign	N
P6	S1	malignant	malignant	Y
	S2	atypical	malignant	N
	S3	malignant	malignant	Y
	S4	atypical	malignant	N
	S5	atypical	malignant	N
	S6	malignant	benign	N
	S7	atypical	benign	Y
P7	S1	atypical	malignant	N
	S2	atypical	malignant	N
	S3	atypical	malignant	N
	S4	atypical	malignant	N
	S5	atypical	benign	Y
	S6	atypical	benign	Y
P8	S1	atypical	malignant	N
	S2	malignant	malignant	Y
	S3	atypical	malignant	N
	S4	atypical	malignant	N
	S5	atypical	malignant	N
	S6	atypical	malignant	N
	S7	atypical	malignant	N

	S8	malignant	malignant	Y
	S9	atypical	benign	Y
	S10	atypical	benign	Y
	S11	atypical	benign	Y
	S12	atypical	benign	Y

Table S3. Consistency of cluster malignancy identification by cytology and scDNA-Seq. Of note, BAL cytology was dichotomized as diagnostic for benign if atypical or benign identified and diagnostic for malignancy if malignancy was identified. A total of 73 single cells from 17 patients (P1-P17) were scrutinized.

Patient No.	Cell cluster No.	# of sequenced CNA profiles	Cytology	scDNA-Seq	Consistency
P1	C1	2	malignant	malignant	Y
	C2	2	malignant	malignant	Y
	C3	2	malignant	malignant	Y
	C4	2	malignant	malignant	Y
	C5	2	malignant	malignant	Y
	C6	1	malignant	malignant	Y
	C7	1	malignant	malignant	Y
	C8	1	atypical	malignant	N
	C9	1	atypical	malignant	N
	C10	1	benign	benign	Y
P2	C1	1	malignant	malignant	Y
	C2	2	malignant	malignant	Y
	C3	3	benign	benign	Y
	C4	1	benign	benign	Y
P3	C1	1	malignant	malignant	Y
	C2	1	malignant	malignant	Y
	C3	1	malignant	malignant	Y
	C4	1	benign	benign	Y
P4	C1	1	malignant	malignant	Y
	C2	1	malignant	malignant	Y
P5	C1	3	benign	malignant	N
	C2	2	benign	benign	Y
	C3	1	benign	benign	Y
	C4	3	benign	benign	Y
	C5	1	benign	benign	Y
P6	C1	2	malignant	malignant	Y
	C2	1	benign	benign	Y
	C3	1	benign	benign	Y
	C4	1	benign	benign	Y
	C5	2	atypical	benign	Y

	C6	1	benign	benign	Y
	C7	1	benign	benign	Y
	C8	1	benign	benign	Y
P7	C1	1	benign	benign	Y
	C2	1	benign	benign	Y
	C3	1	benign	benign	Y
	C4	1	atypical	benign	Y
P8	C1	4	benign	benign	Y
	C2	2	benign	benign	Y
	C3	2	benign	benign	Y
P9	C1	3	malignant	malignant	Y
	C2	1	atypical	benign	Y
P10	C1	2	benign	malignant	N
	C2	1	malignant	benign	N
P11	C1	1	malignant	benign	N
	C2	1	benign	benign	Y
	C3	1	atypical	benign	Y
	C4	1	atypical	benign	Y
P12	C1	1	malignant	malignant	Y
	C2	1	malignant	malignant	Y
	C3	1	malignant	malignant	Y
	C4	1	atypical	benign	Y
	C5	1	malignant	benign	N
P13	C1	1	malignant	malignant	Y
	C2	1	malignant	malignant	Y
P14	C1	1	malignant	malignant	Y
	C2	1	malignant	malignant	Y
	C3	1	malignant	malignant	Y
	C4	1	benign	benign	Y
	C5	1	benign	benign	Y
P15	C1	1	malignant	malignant	Y
	C2	1	atypical	malignant	N
	C3	1	malignant	malignant	Y
P16	C1	1	malignant	malignant	Y
	C2	1	malignant	malignant	Y
	C3	1	malignant	malignant	Y
	C4	1	malignant	malignant	Y
	C5	1	malignant	malignant	Y
	C6	1	malignant	benign	N
	C7	1	atypical	benign	Y
P17	C1	1	malignant	malignant	Y
	C2	1	malignant	malignant	Y
	C3	1	malignant	malignant	Y

Table S4. Demographics and clinical characteristics of patients who were enrolled to generate single-cell training cohorts from BALF specimens for developing LESSEL. LC, lung cancer; N/A, not available; COPD, chronic obstructive pulmonary disease.

Patient #	Age	Sex	Stage	Disease type		Number of CNV-confirmed malignant cells	Number of benign cells
P1	75	M	IB	LC	LUAD	73	/
P2	71	M	IIA	LC	LUAD	30	/
P3	66	F	IIIC	LC	LUAD	77	/
P5	74	F	IIIC	LC	LUAD	21	/
P9	68	M	IV	LC	LUAD	22	/
P6	70	M	IIIB	LC	LUSC	3	/
P7	58	F	IA	LC	LUSC	7	/
P8	68	M	IIB	LC	LUSC	9	/
P10	67	M	IIIA	LC	LUSC	17	/
P18	73	M	IA3	LC	LUSC	17	/
P19	63	M	IA3	LC	LUSC	9	/
P20	60	M	IIIA	LC	LUSC	61	/
P21	66	M	IIIC	LC	LUSC	43	/
P22	70	M	IIIB	LC	LUSC	59	/
P4	75	F	IIIA	LC	SCLC	26	/
P11	80	M	N/A	LC	SCLC	33	/
P23	73	M	IIIA	LC	SCLC	43	/
P24	55	M	IIIC	LC	SCLC	30	
N1	74	M	/	Benign	Infection	/	
N2	71	M	/	Benign	Chronic inflammation	/	
N3	74	M	/	Benign	Tracheobronchial Foreign Body	/	
N4	62	F	/	Benign	Infection	/	
N5	53	M	/	Benign	COPD	/	
N6	60	F	/	Benign	Benign nodule	/	
N7	73	F	/	Benign	Bronchiectasis	/	
N8	61	M	/	Benign	Tuberculosis	/	
N9	34	F	/	Benign	Infection	/	
N10	60	F	/	Benign	Severe pneumonia	/	

Table S5. LESSEL-predicted probabilities of malignant cells in training/validation/test groups (raw data of ROC curves shown in Figure 4D). See separate EXCEL file.

Table S6. Demographics and clinical characteristics of patients who were enrolled to generate external test cohort from Pap-stained fine needle aspiration cytology (FNAC) slides and by membrane-based liquid-based preparation (LBP) method. PSC: pulmonary sarcomatoid carcinoma.

External test cohort							
No	Age	Sex	Subtype	Stage	# of scDNA-Seq confirmed tumor cells	# of LESSEL predicted tumor cells	LESSEL sensitivity (%)
P3	66	F	LUAD	IIIC	20	15	75.00%
P25	53	M	LUAD	IVB	18	8	44.44%
P26	61	M	LUAD	IVA	13	4	30.77%
P27	70	M	SCLC	IIIA	8	5	62.50%
P28	64	M	PSC	IIIB	19	15	78.95%
				Total	78	47	60.26%
				Large-sized cells	55	33	60.00%
				Small-sized cells	23	14	60.90%

	# of LESSEL predicted tumor cells in WSI	# of total cells in WSI	Tumor cell % in WSI analyzed by LESSEL	Calculated tumor cell % in WSI based on LESSEL sensitivity
P3	546	1072	50.93%	67.91%
P25	2543	10554	24.10%	54.21%
P26	184	1250	14.72%	47.84%
P27	713	1552	45.94%	73.51%
P28	1985	3622	54.80%	69.42%

Table S7. Demographics, clinical characteristics and LESSEL-predicted BAL ETC% of patients who performed BAL in the discovery (center 1), validation (center 1) and external validation (center 2) cohorts. See separate EXCEL file.
